# Low-Density Lipoprotein Receptor-Related Protein 1 (LRP1) Is a Negative Regulator of Oligodendrocyte Progenitor Cell Differentiation in the Adult Mouse Brain

**DOI:** 10.3389/fcell.2020.564351

**Published:** 2020-11-13

**Authors:** Loic Auderset, Kimberley A. Pitman, Carlie L. Cullen, Renee E. Pepper, Bruce V. Taylor, Lisa Foa, Kaylene M. Young

**Affiliations:** ^1^Menzies Institute for Medical Research, University of Tasmania, Hobart, TAS, Australia; ^2^School of Medicine, University of Tasmania, Hobart, TAS, Australia

**Keywords:** LRP1, oligodendrocyte, myelin, proliferation, NG2 glia, remyelination, cuprizone, alpha-2-macroglobulin

## Abstract

Low-density lipoprotein receptor-related protein 1 (LRP1) is a large, endocytic cell surface receptor that is highly expressed by oligodendrocyte progenitor cells (OPCs) and LRP1 expression is rapidly downregulated as OPCs differentiate into oligodendrocytes (OLs). We report that the conditional deletion of *Lrp1* from adult mouse OPCs (*Pdgfrα-CreER :: Lrp1^*fl/fl*^*) increases the number of newborn, mature myelinating OLs added to the corpus callosum and motor cortex. As these additional OLs extend a normal number of internodes that are of a normal length, *Lrp1*-deletion increases adult myelination. OPC proliferation is also elevated following *Lrp1* deletion *in vivo*, however, this may be a secondary, homeostatic response to increased OPC differentiation, as our *in vitro* experiments show that LRP1 is a direct negative regulator of OPC differentiation, not proliferation. Deleting *Lrp1* from adult OPCs also increases the number of newborn mature OLs added to the corpus callosum in response to cuprizone-induced demyelination. These data suggest that the selective blockade of LRP1 function on adult OPCs may enhance myelin repair in demyelinating diseases such as multiple sclerosis.

## Graphical Abstract

LRP1 regulates adult oligodendrogenesis and remyelination.

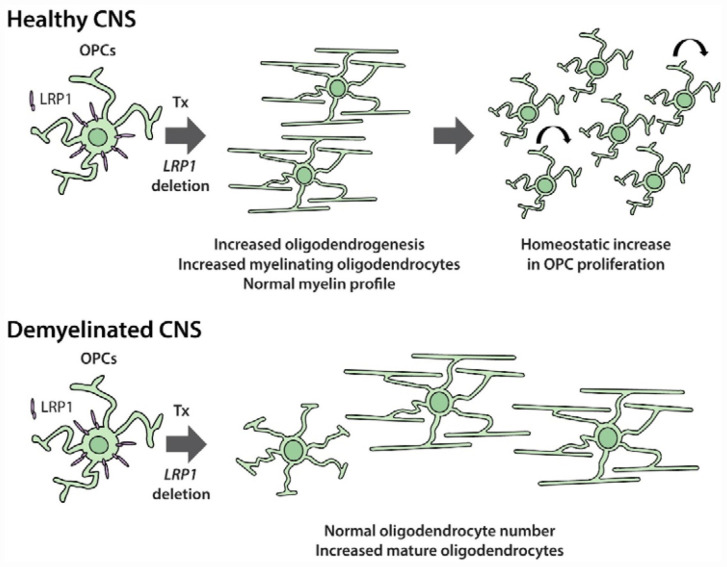

## Introduction

Oligodendrocytes (OLs) myelinate the central nervous system (CNS) to facilitate the saltatory conduction of action potentials and provide essential metabolic support to axons (reviewed by Pepper et al., 2018). The majority of OLs are produced from oligodendrocyte progenitor cells (OPCs) during development, however, adult OPCs also produce new OLs (Dimou et al., 2008; Rivers et al., 2008; Zhu et al., 2008; Kang et al., 2010; Hughes et al., 2013; Young et al., 2013; Hill et al., 2018) that add additional myelin to the mature CNS (Hill et al., 2018; Hughes et al., 2018). A number of signaling pathways have been identified that regulate developmental and adult OPC behavior and oligodendrogenesis, including Notch1 (Genoud et al., 2002; Givogri et al., 2002; Zhang et al., 2009), fibroblast growth factor 2 (Murtie et al., 2005; Zhou et al., 2006; Murcia-Belmonte et al., 2014), mammalian target of rapamycin (Zou et al., 2014; Jiang et al., 2016; Grier et al., 2017) and platelet-derived growth factor A (McKinnon, 2005; Rajasekharan, 2008; Chew et al., 2010) signaling. However, microarray (Cahoy et al., 2008) and RNA sequencing (Zhang et al., 2014; Hrvatin et al., 2018) experiments have uncovered a number of additional genes that are differentially expressed across OL development and have an unknown or partly characterized regulatory function in the OL lineage. One such gene is the *low-density lipoprotein receptor related protein 1* (*Lrp1*).

LRP1, also known as CD91 or the α2 macroglobulin (α2M) receptor, is highly expressed by OPCs and is rapidly downregulated during OL differentiation (Auderset et al., 2016a). This large cell surface receptor, comprising a 515 kDa extracellular α-chain and an 85 kDa β-chain, could influence OPC behavior in a number of ways, as it interacts with a large variety of ligands, as well as extracellular and intracellular proteins, to facilitate signal transduction (Auderset et al., 2016b; Bres and Faissner, 2019). In other cell types, LRP1 acts as a receptor or co-receptor to initiate intracellular signal transduction, but also facilitates ligand endocytosis, transcytosis, or processing (Cam et al., 2005; Parkyn et al., 2008; Su et al., 2008; Yamamoto et al., 2014; Liu et al., 2017; Van Gool et al., 2019; Rauch et al., 2020), as well as receptor, channel and transporter trafficking (Parkyn et al., 2008; Maier et al., 2013; Nakajima et al., 2013; Liu et al., 2015; Boyé et al., 2017; Kadurin et al., 2017; Wujak et al., 2018) to influence blood brain barrier permeability (Polavarapu et al., 2007), lipid metabolism, glucose homeostasis, neuroinflammation (Brifault et al., 2017, 2019; Actis Dato and Chiabrando, 2018) and synaptic plasticity (Zhuo et al., 2000).

*Lrp1* knockout mice are embryonic lethal, as the blastocysts fail to implant (Herz et al., 1992), but the conditional deletion of *Lrp1* from cultured mouse neural stem and progenitor cells (NSPCs) impairs NSPC proliferation and reduces the number of OL lineage cells generated (Hennen et al., 2013; Safina et al., 2016). Furthermore, the conditional deletion of *Lrp1* from *Olig2*^+^ cells (*Olig2-Cre :: Lrp1^*fl/fl*^* mice) impairs oligodendrogenesis in the developing mouse optic nerve, ultimately reducing the proportion of axons that are myelinated and myelin thickness by postnatal day (P)21 (Lin et al., 2017). As ∼20% of optic nerve axons are subsequently myelinated in control and *Lrp1*-deleted mice between P21 and P56, and the discrepancy in g-ratio lessens (Lin et al., 2017), this phenotype may represent a developmental delay in optic nerve myelination. This idea is supported by myelination being grossly normal in the brain and optic nerve of young adult mice following the developmental deletion of *Lrp1* from *Olig1*^+^ cells (Fernandez-Castaneda et al., 2019).

As OPC physiology changes considerably between development and adulthood, and can also differ between CNS regions (Velez-Fort et al., 2010; Pitman et al., 2019; Spitzer et al., 2019), we examined the importance of LRP1 for adult OPC function. The conditional deletion of *Lrp1* from adult OPCs (*Pdgfr*α*-CreER :: Lrp1^*fl/fl*^*) revealed that LRP1 is a negative regulator of oligodendrogenesis in the healthy adult mouse brain. *Lrp1* deletion was associated with an increase in adult OPC proliferation and a significant increase in the number of newborn mature OLs added to the cortex and corpus callosum (see Graphical Abstract). Furthermore, *Lrp1* deletion was associated with a larger number of newborn mature OLs being detected in the corpus callosum of cuprizone fed-mice and less of the corpus callosum showed overt demyelination.

## Materials and Methods

### Transgenic Mice and Their Housing

All animal experiments were approved by the University of Tasmania Animal Ethics (A0016151) and Institutional Biosafety Committees and were carried out in accordance with the Australian code of practice for the care and use of animals for scientific purposes. *Pdgfr*α*-CreER^*T*2^* mice (Rivers et al., 2008) were a kind gift from Prof William D Richardson (University College London). *Pdgfr*α*-CreER^TM^* (Kang et al., 2010; RRID:IMSR_JAX:018280), *Pdgfr*α*-H2BGFP* [*Pdgfr*α*-histGFP* (Hamilton et al., 2003); RRID:IMSR_JAX:007669] and *Lrp1*^*fl/fl*^ (Herz et al., 1992; RRID:IMSR_JAX:012604) mice were purchased from Jackson Laboratories. Cre-sensitive *Rosa26-YFP* (Srinivas et al., 2001; RRID: IMSR_JAX:006148) and *Tau-mGFP* (Hippenmeyer et al., 2005; RRID:IMSR_JAX021162) reporter mice were also purchased from Jackson laboratories. Mice were maintained on a C57BL/6 background and inter-crossed to generate male and female offspring for experimental use. All mice were weaned >P30 to ensure appropriate myelin development, were group housed with same-sex littermates in Optimice micro-isolator cages (Animal Care Systems, CO, United States) and were maintained on a 12-h light/dark cycle at 20°C, with uninhibited access to food and water.

Please note that two distinct *Pdgfr*α*-CreER* transgenic mouse lines were used in this study: the *Pdgfr*α*-CreER^TM^* transgenic mouse line (Kang et al., 2010) was used for the majority of experiments and the lower efficiency (LE) *Pdgfr*α-*CreER*^*T*2^ transgenic mouse line (Rivers et al., 2008), was used to perform the *Tau-mGFP* lineage tracing experiments, as we have previously demonstrated that the *Pdgfr*α*-CreER^TM^* transgenic mouse line cannot be used to induce OPC-specific recombination of the *Tau-mGFP* reporter, despite achieving the OPC-specific recombination of other transgenes (Pitman et al., 2019).

### Genomic DNA Extraction and PCR Amplification

For genotyping, ear biopsies were digested overnight in DNA extraction buffer (100 mM Tris-HCl, 5 mM EDTA, 200 mM NaCl, 0.2% SDS and 120 ng of proteinase k) at 55°C. Cellular and histone proteins were precipitated by incubating samples with 6 M ammonium acetate (Sigma; A1542) on ice and the DNA was precipitated from the supernatant by incubating with isopropyl alcohol (Sigma; I9516). The DNA pellet was washed in 70% ethanol (Sigma; E7023), resuspended in sterile MilliQ water and used as template DNA for polymerase chain reaction (PCR). Each 25 μL reaction contained: 50–100 ng DNA; 0.5 μL of each primer (100 nmol/mL, GeneWorks); 12.5 μL of GoTaq green master mix (Promega) and MilliQ water. The following primers were used: *Lrp1 5′* CATAC CCTCT CAAACC CCTT CCTG and *Lrp1 3′* GCAAG CTCC CTGCTCA GACC TGGA; *Rosa26 wildtype 5′* AAAGT CGCTC TGAGT TGTTAT, *Rosa26 wildtype 3′* GGAGC GGGAG AAATG GATATG and *Rosa26 mutant 5′* GCGAA GAGTT TGTCC TCAACC; *Cre 5*′ CAGGT CTCAG GAGCT ATGTC CAATT TACTG ACCGTA and *Cre 3′* GGTGT TATAAG CAATCC CCAGAA, or *GFP 5′* CCCTG AAGTTC ATCTG CACCAC and *GFP 3′* TTCTC GTTGG GGTCT TTGCTC in a program of: 94°C for 4 min, and 34 cycles of 94°C for 30″, 60°C for 45″ (37 cycles for *Rosa26-YFP* genotyping) and 72°C for 60″, followed by 72°C for 10 min. Following gel electrophoresis [1% (w/v) agarose in TAE containing SYBR-safe (Thermo Fisher Scientific)], the DNA products were visualized using an Image Station 4000 M PRO gel system running Carestream software.

### Tamoxifen Preparation and Administration

Tamoxifen (Sigma) was dissolved in corn oil (Sigma) to a concentration of 40 mg/ml by sonication in a water bath for 2 h at 21°C. Adult mice received tamoxifen (300 mg/kg) daily by oral gavage for 4 consecutive days.

### Tissue Preparation and Immunohistochemistry

Mice were terminally anesthetized with an intraperitoneal (i.p) injection of sodium pentobarbital (30 mg/kg, Ilium) and were transcardially perfused with 4% (w/v) paraformaldehyde (PFA; Sigma) in phosphate buffered saline (PBS). Brains were cut into 2 mm-thick coronal slices using a 1 mm brain matrix (Kent Scientific) before being post-fixed in 4% (w/v) PFA in PBS at 21°C for 90 min. Tissue was cryoprotected in 20% sucrose (Sigma) in PBS and transferred to OCT (Thermo Fisher Scientific) before being snap frozen in liquid nitrogen and stored at −80°C.

Thirty μm coronal brain cryosections were collected and processed as floating sections (as per O’Rourke et al., 2016). Cryosections were exposed to primary antibodies diluted in blocking solution [10% (v/v) fetal calf serum (FCS, Serana) and 0.05% (v/v) triton ×100 in PBS] and incubated overnight at 4°C on an orbital shaker. Primary antibodies included: rabbit anti-LRP1 (1:500, Abcam ab92544; RRID:AB_2234877); goat anti-PDGFRα (1:100, R&D Systems AF1062; RRID:AB_2236879); rabbit anti-ASPA (1:200, Abcam ab97454; RRID:AB_10679051); rabbit anti-LRP2 (1:100, Abcam ab76969, RRID:AB_10673466); rat anti-GFP (1:2000, Nacalai Tesque 04404-26; RRID:AB_2314545); rat anti-MBP (1:100, Millipore MAB386; RRID:AB_94975), rabbit anti-OLIG2 (1:400, Abcam ab9610; RRID:AB_570666); guinea pig anti-IBA1 (1:250, Synaptic Systems 234004; RRID:AB_2493179), and mouse anti-NaBC1 (BCAS1; 1:200, Santa Cruz sc-136342; RRID:AB_10839529). After washing in PBS, cryosections were incubated with secondary antibodies conjugated to Fluors (Life Technologies Corporation), diluted in blocking solution, overnight at 4°C on an orbital shaker. Secondary antibodies included: donkey anti-rat 488 (1:500; RRID: AB_2535794); donkey anti-rabbit 488 (1:1000; RRID: AB_2535792); donkey anti-rabbit 568 (1:1000; RRID: AB_2534017); donkey anti-rabbit 647 (1:1000; RRID: AB_2536183); donkey anti-goat 647 (1:1000; RRID: AB_2535864), donkey anti-mouse 647 (1:1000; RRID AB_162542), or goat anti-guinea pig 488 (1:1000; RRID: AB_2534117). Cell nuclei were visualized by the inclusion of Hoechst 33342 (1:10,000, Invitrogen).

### EdU Administration and Labeling

For the *in vivo* labeling of dividing cells, 5-Ethynly-2′-deoxyuridine (EdU; E10415, Thermo Fisher Scientific) was administered to mice via their drinking water at a concentration of 0.2 mg/ml for up to 21 consecutive days [as per Clarke et al. (2012)]. For *in vitro* labeling, cells were exposed to 2.5 μg/ml EdU in complete OPC medium (see below) for 10 h before the cells were fixed with 4% (w/v) PFA in PBS for 15 min at 21°C. The EdU developing cocktail was prepared according to the AlexaFluor-647 Click-IT EdU kit (Invitrogen) instructions, and brain slices were exposed to the developing reagent for 45 min at 21°C, while coverslips of cultured cells were exposed for 15 min. EdU developing was performed immediately after the secondary antibody was washed from tissue or cells during immunohistochemistry or immunocytochemistry.

### Primary OPC Culture and *in vitro* Gene Deletion

The cortices of P1-10 mice were dissected into Earle’s Buffered Salt Solution (EBSS; Invitrogen, 14155-063), diced into pieces ∼1 mm^3^ and digested in 0.06 mg/ml trypsin (Sigma, T4799) in EBSS at 37°C for 10 min. The trypsin was inactivated by the addition of FCS, before the tissue was resuspended and triturated in EBSS containing 0.12 mg/ml DNAseI (Sigma, 5025). The cell preparation was filtered through a 40 μm sieve (Corning, 352340), centrifuged and resuspended in complete OPC medium [20 ng/ml human PDGF-AA (Peprotech), 10 ng/ml basic fibroblast growth factor (R&D Systems), 10 ng/ml human ciliary neurotrophic factor (Peprotech), 5 μg/ml N-acetyl cysteine (Sigma), 1 ng/ml neurotrophin-3 (Peprotech), 1 ng/ml biotin (Sigma), 10 μM forskolin (Sigma), 1× penicillin/streptomycin (Invitrogen), 2% B27 (Invitrogen), 50 μg/ml insulin (Sigma), 600 ng/ml progesterone (Sigma), 1 mg/ml transferrin (Sigma), 1 mg/ml BSA (Sigma), 400 ng/ml sodium selenite (Sigma) and 160 μg/ml putrescine (Sigma) in DMEM+Glutamax (Invitrogen)]. Cells were plated into 6 well plates coated with >300,000 MW Poly D Lysine (PDL; Sigma, P7405). After 7 DIV, the cells were dislodged by incubating in 1:5 TrypLE (Gibco) in EBSS for ∼10 min at 37°C, before the trypsin was inactivated by the addition of FCS, and cells were collected into EBSS. OPCs were then purified by immunopanning as previously described (Emery and Dugas, 2013). In brief, the cell suspension was transferred to a petri dish pre-coated with anti-PDGFRα (BD Pharmigen 558774; RRID:AB_397117) and the OPCs allowed to adhere for 45 min at 21°C. The non-adherent cells were then removed by rinsing with EBSS and the purified OPCs were stripped by treating with TrypLE diluted 1:5 with EBSS for 5 min at 37°C. The recovered cells were plated onto 13 mm glass coverslips in complete OPC medium.

For experiments where *Lrp1* was deleted *in vitro*, OPCs were plated in complete OPC medium at a density of 20,000 cells per PDL-treated 13 mm coverslip and allowed to settle for 2 days. OPCs were then exposed to 1 μM TAT-Cre (Excellgen, EG-1001) in complete OPC medium at 37°C/5% CO_2_ for 90 min. The TAT-Cre-containing medium was then removed and replaced with fresh complete OPC medium and the cells returned to the incubator for 48 h. To induce differentiation, the complete OPC medium was removed and replaced with OPC differentiation medium [complete OPC medium lacking PDGF-AA and containing 4 μg/ml triiodothyronine (Sigma)] for 4 days before cells were fixed by exposure to 4% PFA (w/v) in PBS for 15 min at 21°C.

LRP1 ligands were reconstituted according to the manufacturer’s instructions. Human recombinant Apolipoprotein E4 (ApoE4; Sigma, A3234) was reconstituted to 0.7 mg/mL in sterile MQ water and was diluted to a concentration of 203 nM in the culture medium. Human recombinant tissue plasminogen activator (tPA; Abcam ab92637) was diluted to a final concentration of 20 nM in the culture medium. Human activated α-2 macroglobulin (^∗^α2M; Sigma, SRP6315) was reconstituted in MilliQ water and diluted to a final concentration of 60 mM in the culture medium.

### Whole Cell Patch Clamp Electrophysiology

Acute coronal brain slices (300 μm) were generated from adult mice carrying the *Pdgfr*α*-histGFP* transgene using a VT1200s vibratome (Leica) as previously described (Pitman et al., 2019). Brain slices were transferred to a bath constantly perfused (2 ml/min) with ∼21°C artificial cerebral spinal fluid (ASCF) containing: 119 mM NaCl, 1.6 mM KCl, 1 mM NaH_2_PO_4_, 26.2 mM NaHCO_3_, 1.4 mM MgCl_2_, 2.4 mM CaCl_2_, and 11 mM glucose (300 ± 5 mOsm/kg), saturated with 95% O_2_/5% CO_2_, Whole cell patch clamp recordings of GFP^+^ cells in the motor cortex were collected using a HEKA Patch Clamp EPC800 amplifier and pCLAMP 10.5 software (Molecular devices; RRID: SCR_011323).

To record AMPA (α-amino-3-hydroxy-5-methyl-4-isoxazolepropionic acid)/kainate receptor currents, recording electrodes (3–6 MΩ) were filled with an internal solution containing: 125 mM Cs-methanesulfonate, 5 mM TEA-Cl, 2 mM MgCl_2_, 8 mM HEPES, 9 mM EGTA, 10 mM phosphocreatine, 5 mM MgATP, and 1 mM Na_2_GTP, and set to a pH of 7.2 with CsOH and an osmolarity of 290 ± 5 mOsm/kg. Upon breakthrough, cells were held at −50 mV and a series of voltage steps (up to+30 mV) applied to determine the presence of a voltage-gated sodium channel current. GFP^+^ cells with a voltage-gated sodium current >100 pA were considered OPCs. All subsequent recordings were undertaken in ACSF containing 50 μm (2R)-amino-5-phosphonovaleric acid (APV; Sigma) and 1 μM tetrodotoxin (TTX; Sigma). Cells were held at −60 mV and currents elicited by applying 200 ms voltage steps from −80 to 20 mV (20 mV increments). After taking baseline recordings, currents were then elicited in ACSF containing 100 μM kainate. The mean steady state current (last 100 ms) of each voltage step was measured.

Voltage gated calcium channel (VGCC) current recordings were made using solutions previously described (Pitman et al., 2019). All other voltage gated currents (potassium and sodium) were blocked. To record *L*-type VGCC currents, OPCs were held at −50 mV and a series of 500 ms voltage steps (−60 to+30 mV) applied using a P/N subtraction protocol. The current-density relationship is presented as the average steady state current (the last 100 ms of the voltage steps) from ∼3 recordings per cell. To elicit currents through *T*-type VGCCs, OPCs were held at −50 mV and the cell was hyperpolarized to −120 mV for 200 ms before applying voltage steps from −70 to 30 mV (Fulton et al., 2010; Haberlandt et al., 2011). The maximum amplitude of the fast, transient inward current, revealed by the brief hyperpolarization, was measured from ∼3 recordings per cell.

Access resistance was measured before and after all recordings and an access resistance >20 MΩ resulted in exclusion of that recording. Due to the high membrane resistance of OPCs (>1 GΩ) during VGCC current recordings, recordings were made without series resistance compensation. However, series resistance compensation was applied for AMPA current recordings (60–80%). Measurements were made from each data file using Clampfit 10.5.

### Cuprizone Administration and Black-Gold Myelin Staining

Mice were transferred onto a diet of crushed mouse food (Barrestock) containing 0.2% (w/w) cuprizone powder (C9012, Sigma), which was refreshed every 2 days for 5 weeks. Mice were perfusion fixed, their tissue processed as described above and 30 μm coronal brain floating cryosections collected into PBS. Cryosections were transferred onto glass microscope slides (Superfrost) and allowed to dry before being rehydrated in MilliQ water for 3 min and incubated with preheated 0.3% black-gold II stain (Millipore, AG105) at 60°C for 30 min. Slides were washed twice with MilliQ water before being incubated with preheated 1% (v/v) sodium thiosulphate solution at 60°C for 3 min, washed in MilliQ water (3 × 2 min), dehydrated using a series of graded alcohol steps, incubated in xylene (Sigma, 214736) for 3 min, and mounted with DPX mounting medium (Sigma, 06522).

### Microscopy and Statistical Analyses

Fluorescent labeling was visualized using an UltraView Spinning Disk confocal microscope with Volocity software (Perkin Elmer, Waltham, United States). The motor cortex and corpus callosum were defined as regions of interest using anatomical markers identified in the Allen Mouse Brain Atlas, in brain sections collected between Bregma level 1.10 and −0.10 mm. Confocal images were collected using standard excitation and emission filters for DAPI, FITC (Alexa Fluor-488), TRITC (Alexa Fluor-568) and CY5 (Alexa Fluor-647). To quantify cell density, or the proportion of cells that proliferate or differentiate, a 10× or 20× air objective was used to collect images with 2 μm *z*-spacing that spanned the defined region of interest within a brain section. These images were stitched together using Volocity software to create a single image of that region for analysis. A minimum of 3 brain sections were imaged per mouse. To quantify oligodendrocyte morphology and measure myelin internodes a 40× (air) or 60× (water) objective was used to collect images with 1 μm *z*-spacing of individual mGFP^+^ OLs or single fields of view containing internodes within each region of interest. Black-gold myelin staining was imaged using a light microscope with a 2.5× objective and images were manually stitched together using Adobe Photoshop CS6 to recreate the region of interest. Cell counts were performed by manually evaluating the labeling of individual cells and area measurements were made by manually defining the region of interest within Photoshop CS6 (Adobe, San Jose, United States) or Image J (NIH, Bethesda, MD, United States). All measurements were made blind to the experimental group and treatment conditions.

Statistical comparisons were made using GraphPad Prism 8.0 (La Jolla, CA, United States; RRID: SCR_002798). Data were first assessed using the D’Agostino-Pearson normality test. Data that were normally distributed were analyzed by a parametric test [*t*-test, one-way analysis of variance (ANOVA) or two-way ANOVA for group comparisons with a Bonferroni *post hoc* test], and data that were not normally distributed were analyzed using a Mann–Whitney *U* test or Kolmogorov–Smirnov test. Lesion size (black-gold staining) data were analyzed using a *t*-test with a Welch’s correction to account for the uneven variance between groups. Data sets with *n* = 3 in any group were analyzed using parametric tests, as the non-parametric equivalents rely on ranking and are unreliable for small sample sizes (GraphPad Prism 8.0). To determine the rate at which OPCs become labeled with EdU over time, these data were analyzed by performing linear regression analyses. Details of the statistical comparisons are provided in each figure legend or in text when the data are not presented graphically. Statistical significance was defined as *p* < 0.05.

## Results

### Tamoxifen Administration Deletes *Lrp1* From Essentially All OPCs in the Adult Mouse Brain

**FIGURE 1:**
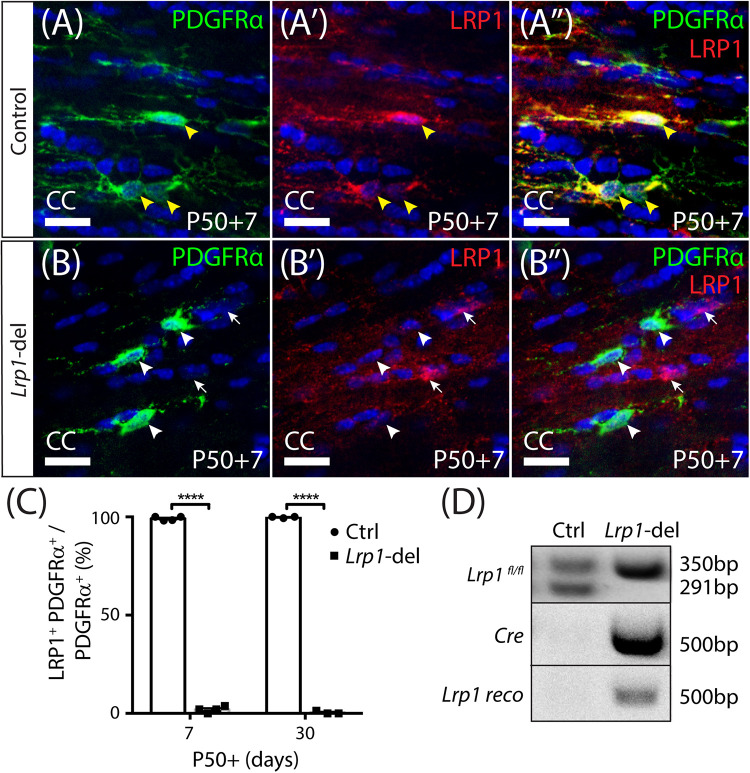
*Lrp1* can be conditionally deleted from adult OPCs at high efficiency. Coronal brain sections from P57+7 and P57+30 control (*Pdgfr*α*-CreER^*TM*^) and Lrp1*-deleted (*Pdgfr*α*-CreER^*TM*^ :: Lrp1^*fl/fl*^*) mice were immunolabeled to detect OPCs (PDGFRα, green), LRP1 (red), and cell nuclei (Hoechst 33342, blue). **(A–A″)** Compressed *z*-stack confocal image of LRP1^+^ OPCs (solid yellow arrow heads) in the corpus callosum (CC) of a P50+7 control mouse. **(B–B″)** Compressed *z*-stack confocal image of LRP1-neg OPCs (solid white arrow heads) in the CC of a P50+7 *Lrp1*-deleted mouse. White arrows indicate PDGFRα-neg cells that remain LRP1^+^ in the *Lrp1-*deleted mice. **(C)** The proportion (%) of PDGFRα^+^ OPCs that express LRP1 in P50+7 and P50+30 control and *Lrp1*-deleted mice [mean ± SD for *n* ≥ 3 mice per genotype per time-point; 2-way ANOVA: *Genotype F* (1,10) = 2.8, *p* < 0.0001; *Time F* (1,10) = 0.52, *p* = 0.5; *Interaction F* (1,10) = 3.44, *p* = 0.09]. Bonferroni multiple comparisons ****p ≤ 0.0001. **(D)** PCR amplification of genomic DNA extracted from the brain of a P50+7 control (*Lrp1*^*fl/+*^) and *Lrp1*-deleted (*Pdgfr*α*-CreER^*TM*^ :: Lrp1^*fl/fl*^*) mouse indicates that recombination, producing the *Lrp1* reco band, only occurs in *Lrp1*-deleted mice. Scale bars represent 17 μm.

To determine the role that LRP1 plays in regulating adult myelination, *Lrp1* was conditionally deleted from OPCs in young adult mice. Tamoxifen was administered to P50 control (*Lrp1*^*fl/fl*^) and *Lrp1*-deleted (*Pdgfr*α*-CreER^TM^ :: Lrp1^*fl/fl*^*) mice and brain tissue examined 7 or 30 days later (at P50+7 and P50+30, respectively). Coronal brain cryosections from control (Figure 1A) and *Lrp1*-deleted mice (Figure 1B) were immunolabeled to detect LRP1 (red) and OPCs (PDGFRα, green). Consistent with our previous findings (Auderset et al., 2016a), essentially all OPCs in the corpus callosum of control mice expressed LRP1 (Figure 1C; P50+7: 99 ± 0.6%, mean ± SD for *n* = 4 mice; P50+30: 99.7 ± 0.3%, mean ± SD for *n* = 3 mice). However, in the corpus callosum of P50+7 *Lrp1-*deleted mice, only 2 ± 0.8% of PDGFRα^+^ OPCs expressed LRP1 (mean ± SD for *n* = 4 mice) and by P50+30, only 0.5 ± 0.5% of OPCs expressed LRP1 (mean ± SD for *n* = 3 mice; Figure 1C), confirming the successful deletion of *Lrp1* from adult OPCs. Similarly, in the motor cortex of P50+7 control mice, 100 ± 0% of PDGFRα^+^ OPCs expressed LRP1 while only 0.4 ± 0.4% of PDGFRα^+^ OPCs expressed LRP1 in the motor cortex of *Lrp1*-deleted mice (mean ± SD for *n* = 4 mice per genotype). Other brain cell types that express LRP1, such as neurons and astrocytes, retained their expression of LRP1 (e.g., white arrows in Figure 1B). As the Cre-mediated recombination of the floxed *Lrp1* gene deletes the extracellular coding region of *Lrp1*, recombination was also confirmed by performing a PCR analysis of genomic DNA from the brains of control (*Lrp1*^*fl*/ +^) and *Lrp1-*deleted (*Pdgfr*α*-CreER^TM^ :: Lrp1^*fl/fl*^*) mice after tamoxifen treatment. *Lrp1-*deletion enabled the amplification of a recombination-specific DNA product from *Lrp1*-deleted brain DNA that was not amplified from control brain DNA (Figure 1D). These data confirm that tamoxifen administration to *Pdgfr*α*-CreER^TM^ :: Lrp1^*fl/fl*^* transgenic mice efficiently and specifically deletes *Lrp1* from adult OPCs.

### *Lrp1* Deletion Produces a Delayed Increase in Adult OPC Proliferation

**FIGURE 2:**
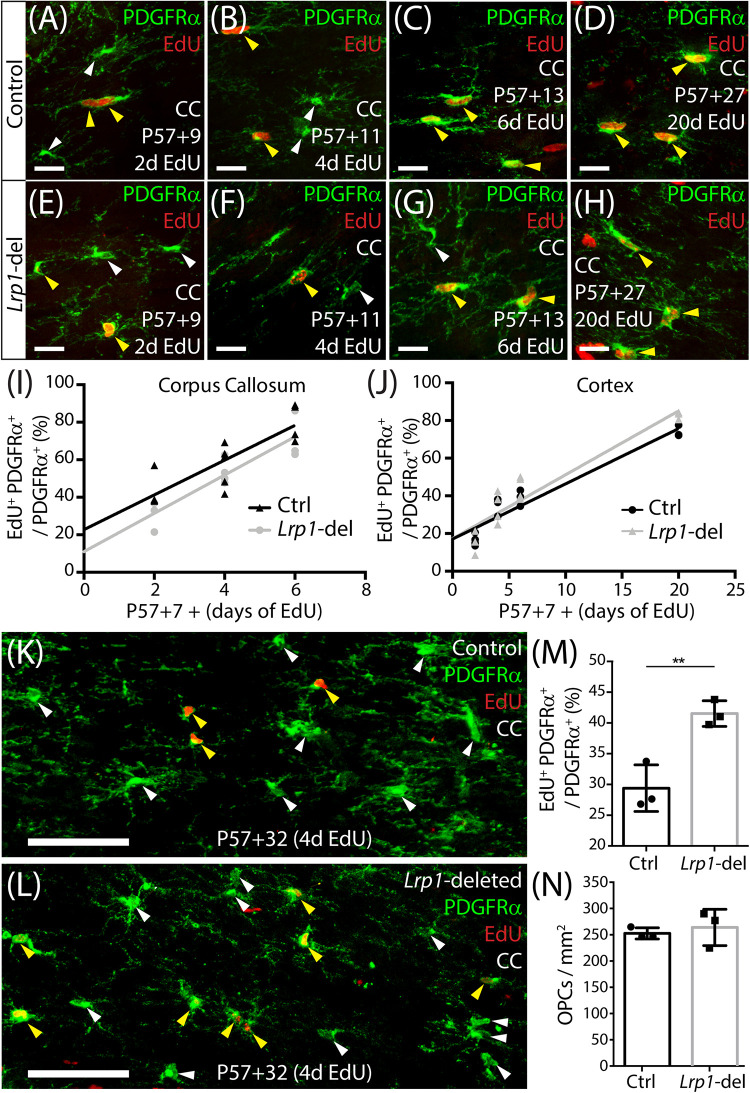
Adult OPC proliferation increases following *Lrp1* deletion. **(A–H)** Compressed confocal *z*-stacks from the corpus callosum (CC) of control (*Pdgfr*α*-CreER^*TM*^*) and *Lrp1-*deleted (*Pdgfr*α*-CreER^*TM*^ :: Lrp1^*fl/fl*^)* mice, immunolabeled to detect OPCs (PDGFRα, green) and EdU (red) after 2, 4, 6, or 20 days of EdU delivery. **(I)** Graph of the proportion (%) of OPCs that incorporate EdU, after 2, 4, or 6 days of delivery, in the CC of control (black) and *Lrp1*-deleted (gray) mice (*n* ≥ 3 mice per genotype per time-point). The rate of EdU uptake was unaffected by genotype (*p* = 0.7; linear regression for controls: *m* = 9.2 ± 1.8% per day and *R*^2^ = 0.7; linear regression for *Lrp1*-deleted: *m* = 10.2 ± 1.8% per day and *R*^2^ = 0.8). **(J)** Graph of the proportion (%) of OPCs that incorporate EdU, after 2, 4, 6, or 20 days of delivery, in the motor cortex of control (black) and *Lrp1*-deleted (gray) mice (*n* ≥ 3 mice per genotype per time-point). The rate of EdU uptake was unaffected by genotype (*p* = 0.3; linear regression for control: *m* = 2.9 ± 0.3 cells per day and *R*^2^ = 0.9; linear regression for *Lrp1*-deleted: *m* = 3.4 ± 0.3 cells per day and *R*^2^ = 0.9). **(K,L)** Compressed confocal *z*-stacks from the CC of P57+32 control and *Lrp1*-deleted mice that received EdU via the drinking water for 4 consecutive days (from P57+28), and were immunolabeled to detect OPCs (PDGFRα, green) and EdU (red). **(M)** The proportion (%) of OPCs that were EdU labeled in the CC of P57+32 control mice (black) and *Lrp1*-deleted mice (gray) that received 4 days of EdU labeling (mean ± SD, *n* = 3 mice per genotype; unpaired *t*-test, **p = 0.008). **(N)** Quantification of the density of OPCs in the CC of P57+32 control mice (black) and *Lrp1*-deleted mice (gray) (mean ± SD, *n* = 3 mice per genotype; unpaired *t*-test, *p* = 0.6). Solid white arrow heads indicate EdU-neg OPCs. Solid yellow arrowheads indicate EdU^+^ OPCs. Scale bars represent 17 μm **(A–H)** or 70 μm **(K,L)**.

Oligodendrocyte progenitor cells divide more frequently in white than gray matter regions of the adult mouse CNS (Psachoulia et al., 2009) and their homeostatic proliferation ensures that a stable pool of progenitors is maintained (Hughes et al., 2013). To determine whether LRP1 regulates the rate that adult OPCs re-enter the cell cycle to divide or the fraction of adult OPCs that proliferate, we delivered a thymidine analog, EdU, to P57+7 control and *Lrp1*-deleted mice via their drinking water for 2, 4, 6, or 20 days. Coronal brain cryosections from control (Figures 2A–D) and *Lrp1*-deleted (Figures 2E–H) mice were processed to detect PDGFRα^+^ OPCs (green) and EdU (red). When quantifying the proportion of OPCs that became EdU labeled over time, we found that 20 days of EdU-delivery resulted in EdU uptake by all OPCs in the corpus callosum of control and *Lrp1*-deleted mice (100 ± 0 and 100 ± 0%, respectively), indicating that the proportion of OPCs that can proliferate is not influenced by LRP1 signaling. Furthermore, the rate of EdU incorporation was equivalent for OPCs in the corpus callosum of control and *Lrp1*-deleted mice (Figure 2I), suggesting that LRP1 does not influence the rate at which OPCs enter or transition through the cell cycle to become EdU-labeled. OPCs in the motor cortex incorporated EdU at a slower rate than those in the corpus callosum (compare the slope of the regression lines in Figures 2I,J), but OPC proliferation in the motor cortex was also unaffected by *Lrp1*-deletion (Figure 2J).

While the loss of LRP1 did not immediately influence OPC proliferation, LRP1 may regulate receptor and channel recycling at the cell membrane (Takayama et al., 2005; Liu et al., 2010; Pi et al., 2012; Maier et al., 2013; Nakajima et al., 2013), such that *Lrp1*-deletion may not immediately perturb OPC behavior. To explore this possibility, we delivered tamoxifen to young adult (P57) control and *Lrp1*-deleted mice and waited 28 days before administering EdU via the drinking water for 4 consecutive days. Coronal brain cryosections from P57+32 control (Figure 2K) and *Lrp1*-deleted (Figure 2L) mice were processed to detect PDGFRα^+^ OPCs (green) and EdU (red). The proportion of OPCs that incorporated EdU over the 4-day labeling period was significantly higher in the corpus callosum of *Lrp1*-deleted mice than controls (Figure 2M). This was also true in the motor cortex, where 15.0 ± 1.02% of control and 20.1 ± 2.53% of *Lrp1*-deleted OPCs had incorporated EdU (mean ± SD for *n* = 3 mice per genotype; unpaired *t*-test, *p* = 0.03). This increase in OPC proliferation was not accompanied by a change in the density of PDGFRα^+^ OPCs (Figure 2N), suggesting that the additional cells must either differentiate or die.

### *Lrp1* Deletion Increases the Number of New OLs Added to the Adult Mouse Brain

To determine whether LRP1 regulates OL production by adult OPCs, tamoxifen was given to P57 control (*Pdgfr*α*-CreER^TM^ :: Rosa26-YFP)* and *Lrp1*-deleted (*Pdgfr*α*-CreER^TM^ :: Rosa26-YFP :: Lrp1^*fl/fl*^*) mice to fluorescently label adult OPCs and the new OLs they produce. At P57+14, coronal brain cryosections were immunolabeled to detect YFP (green), PDGFRα (red), and OLIG2 (blue) and confirm the specificity of labeling (Supplementary Figure 1). Consistent with our previous findings in control mice (O’Rourke et al., 2016), all YFP^+^ cells in the corpus callosum of control and *Lrp1*-deleted mice were either PDGFRα^+^ OLIG2^+^ OPCs or PDGFRα-negative OLIG2^+^ newborn OLs (Supplementary Figure 1). In the motor cortex, the vast majority of YFP^+^ cells expressed OLIG2 (control: 96.2 ± 0.91%; *Lrp1*-deleted: 94.3 ± 1.02%, mean ± SD for *n* = 3 mice per genotype; Supplementary Figure 1). The small number of YFP^+^ OLIG2-negative cells identified in the cortex had the morphological characteristics of neurons, consistent with previous reports that the *Pdgfr*α promoter is active in a small subset of cortical neurons (Clarke et al., 2012). These cells were excluded from all subsequent analyses.

**FIGURE 3:**
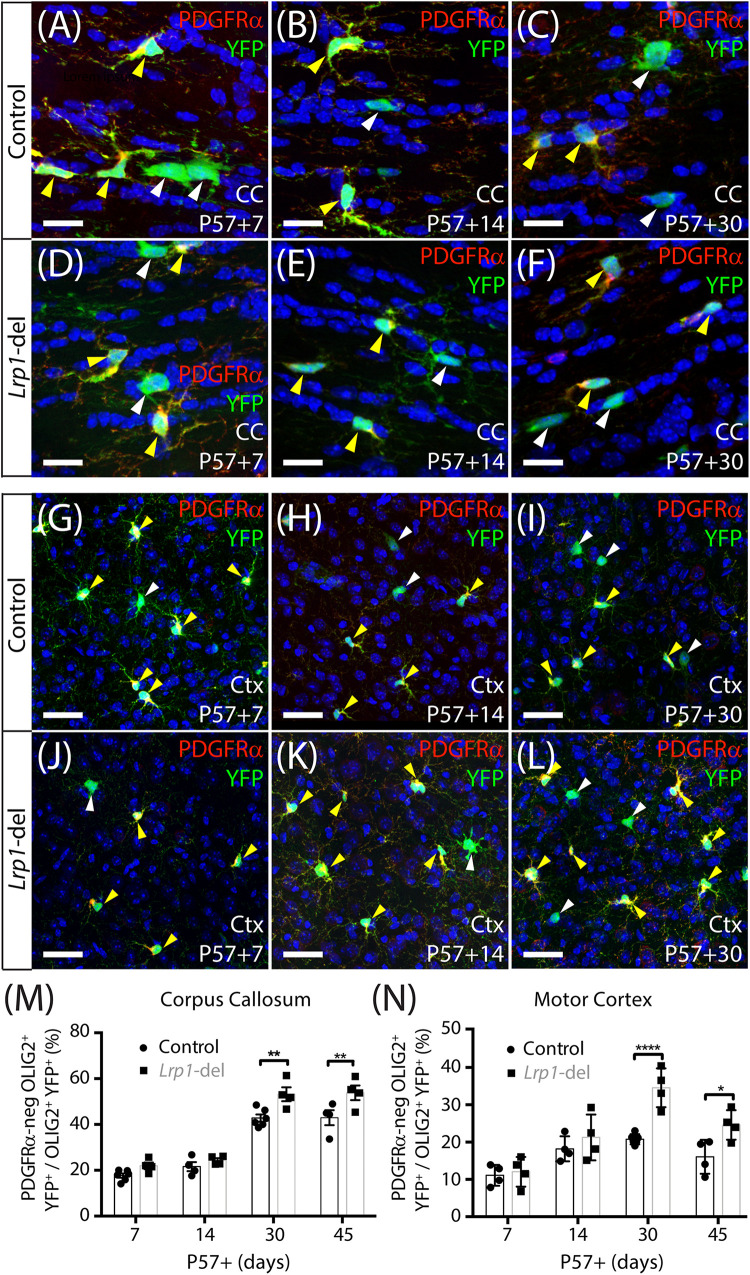
*Lrp1* deletion increases the number of new OLs added to the adult mouse corpus callosum and motor cortex. **(A–L)** Confocal images of the corpus callosum (CC; **A–F**) and motor cortex (Ctx; **G–L**) of P57+7, P57+14, and P57+30 control (*Pdgfr*α*-CreER^*TM*^* :: *Rosa26-YFP*) and *Lrp1*-deleted (*Pdgfr*α*-CreER^*TM*^ :: Rosa26-YFP :: Lrp1^*fl/fl*^*) mice immunolabeled to detect PDGFRα (red), YFP (green), and the nuclear marker Hoechst 33342 (blue). Solid yellow arrowheads indicate YFP^+^ PDGFRα^+^ OPCs. Solid white arrowheads indicate YFP^+^ PDGFRα-neg newborn OLs. **(M)** Graph of the proportion (%) of YFP^+^ cells that are YFP^+^ PDGFRα-neg OLIG2^+^ newborn OLs in the CC of control and *Lrp1-*deleted mice [mean ± SD for *n* ≥ 4 mice per genotype per time-point; 2-way ANOVA: *Genotype F* (1, 28) = 22.3, *p* < 0.0001; *Time F* (3, 28) = 109.7, *p* < 0.0001; *Interaction F* (3, 28) = 1.9, *p* = 0.15]. **(N)** Graph of the proportion (%) of YFP^+^ cells that are YFP^+^ PDGFRα-neg OLIG2^+^ newborn OLs in the motor cortex of control and *Lrp1-*deleted mice [mean ± SD for *n* ≥ 4 mice per genotype per time-point; 2-way ANOVA: *Genotype F* (1, 26) = *22.5, p* < *0.0001; Time F* (3, 26) = *23.4, p* < *0.0001; Interaction F* (3, 26) = *4.56, p* = *0.011].* Bonferroni multiple comparisons: *p < 0.05, **p < 0.01, ****p < 0.0001. Scale bars represent 17 μm **(A–F)** and 34 μm **(G–L)**.

**FIGURE 4:**
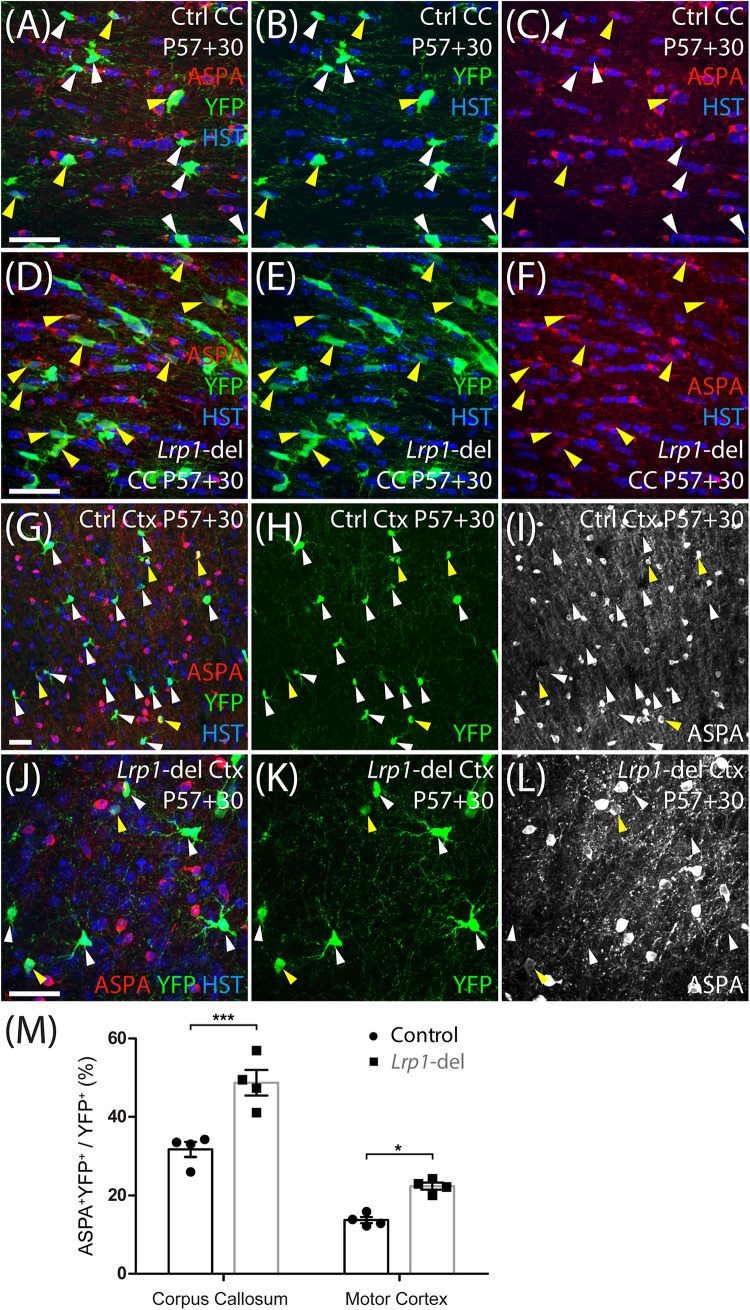
*Lrp1* deletion increases the number of newborn ASPA^+^ OLs in the adult mouse corpus callosum and motor cortex. **(A–L)** Confocal images from the corpus callosum (CC; **A–F**) or motor cortex (Ctx; **G–L**) of P57+30 control (*Pdgfr*α*-CreER^*TM*^:: Rosa26-YFP*) and *Lrp1*-deleted (*Pdgfr*α*-CreER^*TM*^:: Rosa26-YFP:: Lrp1^*fl/fl*^*) mice that were immunolabeled to detect ASPA (red), YFP (green), and the nuclear marker Hoechst 33342 (HST, blue). Solid yellow arrowheads indicate YFP^+^ ASPA^+^ mature OLs. Solid white arrowheads indicate YFP^+^ ASPA-neg cells. **(M)** Quantification of the proportion (%) of YFP^+^ cells that are YFP^+^ ASPA^+^ newborn mature OLs in the CC and Ctx of P57+30 control and *Lrp1-*deleted mice [mean ± SD for *n* = 4 mice per genotype; 2-way ANOVA: *Genotype F* (1, 12) = 125.3, *p* < 0.0001; *Brain region F* (1, 12) = 42.1, *p* < 0.0001; *Interaction F* (3, 28) = 4.5, *p* = 0.056]. Bonferroni multiple comparisons: *p < 0.05, ***p < 0.001. Scale bars represent 30 μm **(A–J)**.

To determine whether LRP1 influences oligodendrogenesis, we quantified the proportion of YFP^+^ cells that were PDGFRα-negative OLIG2^+^ newborn OLs in the corpus callosum (Figures 3A–F) or motor cortex (Figures 3G–L) of P57+7, P57+14, P57+30 and P57+45 control and *Lrp1*-deleted mice. At P57+7 and P57+14 oligodendrogenesis was equivalent in the corpus callosum of control and *Lrp1*-deleted mice, however, by P57+30 a larger proportion of YFP^+^ cells had become newborn OLs in the corpus callosum of *Lrp1*-deleted mice, and this effect was sustained at P57+45 (Figure 3M). Similarly, for the first 2 weeks, OL production was equivalent for OPCs in the motor cortex of control and *Lrp1*-deleted mice, however, by P57+30, the proportion of YFP^+^ cells that were newborn OLs was higher in the motor cortex of *Lrp1*-deleted mice than controls (Figure 3N). This produced a corresponding increase in the density of new OLs detected in the corpus callosum (control: 107.2 ± 14.9 cells/mm^2^; *Lrp1*-deleted: 161.8 ± 27.4 cells/mm^2^; mean ± SD, *n* = 7 control and *n* = 4 *Lrp1*-deleted mice; *t*-test, *p* = 0.0005) and motor cortex (control: 42.55 ± 9.2 cells/mm^2^; *Lrp1*-del: 61.34 ± 7.0 cells/mm^2^; mean ± SD, *n* = 4 mice per genotype; *t*-test, *p* = 0.004) of P57+30 *Lrp1*-deleted mice compared to controls. By performing immunohistochemistry to detect YFP (green) and the mature OL marker, aspartoacylase (ASPA, red) (Figures 4A–L), we confirmed that *Lrp1*-deletion increased the number of newborn mature OLs added to the adult mouse corpus callosum and motor cortex (Figure 4M).

### Myelinating OLs Produced by *Lrp1*-Deleted OPCs Elaborate a Normal Number of Internodes

**FIGURE 5:**
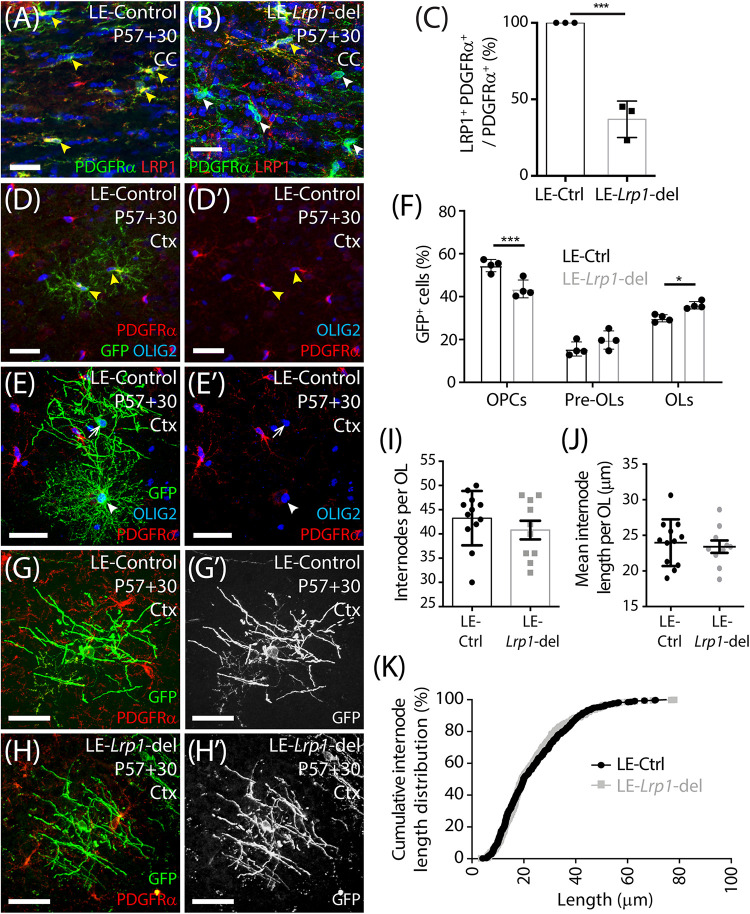
*Lrp1* deletion increases the number of mature, myelinating OLs added to the motor cortex of adult mice. **(A,B)** Compressed confocal *z*-stack from the corpus callosum (CC) of P57+30 LE-control (*Pdgfr*α*-CreER^*T*2^*) and LE-*Lrp1*-deleted (*Pdgfr*α*-CreER^*T*2^ :: Lrp1^*fl/fl*^)* mice that were immunolabeled to detect OPCs (PDGFRα, green), LRP1 (red), and Hoechst 33342 (blue). Solid yellow arrowheads indicate OPCs that express LRP1. Solid white arrowheads indicate OPCs that do not express LRP1. **(C)** The proportion (%) of PDGFRα^+^ OPCs in the CC of LE-control and the LE-*Lrp1*-deleted mice that express LRP1 (mean ± SD for *n* = 3 mice per group; unpaired *t*-test, ***p < 0.001). **(D–E′)** Compressed confocal *z*-stack from the motor cortex (Ctx) of a P57+30 control (LE-*Pdgfr*α*-CreER^*T*2^ :: Tau-mGFP*) mouse immunolabeled to detect PDGFRα (red), GFP (green), and OLIG2 (blue). Solid yellow arrowheads indicate GFP^+^ PDGFRα^+^ OLIG2^+^ OPCs. Solid white arrowhead indicates a GFP^+^ PDGFRα-neg OLIG2^+^ newborn pre-myelinating OL. The white arrow indicates a GFP^+^ PDGFRα-neg OLIG2^+^ newborn myelinating OL. **(F)** Quantification of the proportion (%) of GFP^+^ cells that are PDGFRα^+^ OLIG2^+^ OPCs, PDGFRα-neg OLIG2^+^ premyelinating OLs (pre-OLs) and PDGFRα-neg OLIG2^+^ myelinating OLs (OLs) [mean ± SD for *n* = 4 mice per genotype; 2-way ANOVA: *Maturation stage F* (2,18) = *195.1, p* < *0.0001; Genotype F* (1, 18) = *0.032, p* = *0.85; Interaction F* (2, 18) = *17.1, p < 0.0001*]. Bonferroni multiple comparisons: *p = 0.046 and ***p = 0.0004. **(G–H′)** Compressed confocal *z*-stack showing GFP^+^ PDGFRα-neg myelinating OLs in the motor cortex of P57+30 LE-control and LE-*Lrp1*-deleted mice. **(I)** The number of internodes elaborated by individual GFP^+^ myelinating OLs in the motor cortex of LE-control and LE-*Lrp1*-deleted mice (mean ± SEM for *n* ≥ 10 OLs from *n* = 3 mice per genotype; Mann–Whitney Test, *p* = 0.38). **(J)** The average length of internodes elaborated by individual GFP^+^ myelinating OLs in LE-control and LE-*Lrp1*-deleted mice (mean ± SEM for *n* ≥ 10 OLs from *n* = 3 mice per genotype; unpaired *t*-test, *p* = 0.67). **(K)** Cumulative length distribution plot for GFP^+^ internodes measured in the motor cortex of P57+30 LE-control and LE-*Lrp1*-deleted mice (*n* = 519 LE-control GFP^+^ internodes and *n* = 408 LE-*Lrp1-deleted* GFP^+^ internodes measured from *n* = 3 mice per genotype; K-S test, *D* = 0.053, *p* = 0.5). Scale bars represent 34 μm **(A,B)** or 17 μm **(G,H)**.

As OPCs differentiate, they rapidly downregulate their expression of PDGFRα, the NG2 proteoglycan and voltage-gated sodium channels (NaV) (Richardson et al., 1988; Pringle et al., 1989; De Biase et al., 2010; Kukley et al., 2010; Clarke et al., 2012). They become highly ramified pre-myelinating OLs that either die or continue to mature into myelinating OLs and elaborate myelin internodes (Trapp et al., 1997; Psachoulia et al., 2009; Hughes et al., 2013; Young et al., 2013; Tripathi et al., 2017; Pitman et al., 2019). To grossly examine the myelin profile of adult-born OLs in control and *Lrp1*-deleted mice, we fluorescently labeled a subset of OPCs in the adult mouse brain with a membrane-targeted form of green fluorescent protein (GFP). We have previously shown that tamoxifen delivery to adult *Pdgfr*α*-CreER^TM^ :: Tau-GFP* mice does not result in the specific fluorescent labeling of OPCs and their progeny (Pitman et al., 2019). Therefore, for this experiment, we instead delivered tamoxifen to adult LE-control (*Pdgfr*α*-CreER^*T*2^ :: Tau-GFP*) and LE-*Lrp1*-deleted (*Pdgfr*α*-CreER^*T*2^ :: Tau-GFP :: Lrp1^*fl/fl*^*) mice. The *Pdgfr*α*-CreER^*T*2^* transgenic mouse (Rivers et al., 2008) has a lower recombination efficiency (LE) than the *Pdgfr*α*-CreER^TM^* transgenic mouse (Kang et al., 2010), so we first evaluated the efficiency of *Lrp1* deletion using this mouse model. Coronal brain cryosections from P57+30 LE-control and LE-*Lrp1*-deleted mice were immunolabeled to detect PDGFRα and LRP1 (Figures 5A,B). While 100 ± 0% of PDGFRα^+^ OPCs expressed LRP1 in the motor cortex of LE-control mice, only 35 ± 9% of PDGFRα^+^ OPCs expressed LRP1 in the motor cortex of LE-*Lrp1*-deleted mice (mean ± SD for *n* = 3 mice per genotype). The recombination efficiency was similar in the corpus callosum with 100 ± 0% of OPCs expressing LRP1 in LE-control mice and only 37 ± 7% in LE-*Lrp1*-deleted mice (Figure 5C).

Only ∼65% of OPCs lacked LRP1 in the LE-*Lrp1*-deleted mice, but this was sufficient to increase the number of newborn OLs detected in the brain. When P57+30 LE-control and LE-*Lrp1*-deleted mouse brain cryosections were immunolabeled to detect GFP (green), PDGFRα (red) and OLIG2 (blue) (Figures 5D,E), we found that the proportion of GFP^+^ cells that became PDGFRα-negative OLIG2^+^ newborn OLs was significantly elevated in the motor cortex of LE-*Lrp1*-deleted mice (56.3 ± 2.06%) compared to control mice (49.2 ± 1.51%, mean ± SD for *n* = 4 mice per genotype; unpaired *t*-test *p* = 0.03). Using the membrane targeted GFP to further subdivide the newborn OLs into premyelinating and myelinating OLs, we determined that *Lrp1* deletion significantly increased the proportion of GFP^+^ cells that had become myelinating OLs (Figure 5F).

Despite the difference in overall cell number, the morphology of the myelinating OLs added to the brain of control and *Lrp1*-deleted mice was equivalent (Figures 5G,H). Our detailed morphological analysis of individual GFP^+^ myelinating OLs in the motor cortex of LE-control and LE-*Lrp1*-deleted mice revealed that neither the average number of internodes elaborated by GFP^+^ myelinating OLs (Figure 5I) or the mean length of internodes elaborated by GFP^+^ myelinating OLs (Figure 5J) was changed by *Lrp1* deletion. Additionally, LRP1 expression did not influence the length distribution for internodes elaborated by newborn myelinating OLs in the motor cortex (Figure 5K). These data indicate that LRP1 negatively regulates the number of myelinating OLs added to the healthy adult mouse brain but does not influence their gross myelination profile.

### *Lrp1* Deletion Does Not Influence NaV, AMPA Receptor, *L*- or *T*-Type VGCC, PDGFRα or LRP2 Expression by OPCs

**FIGURE 6:**
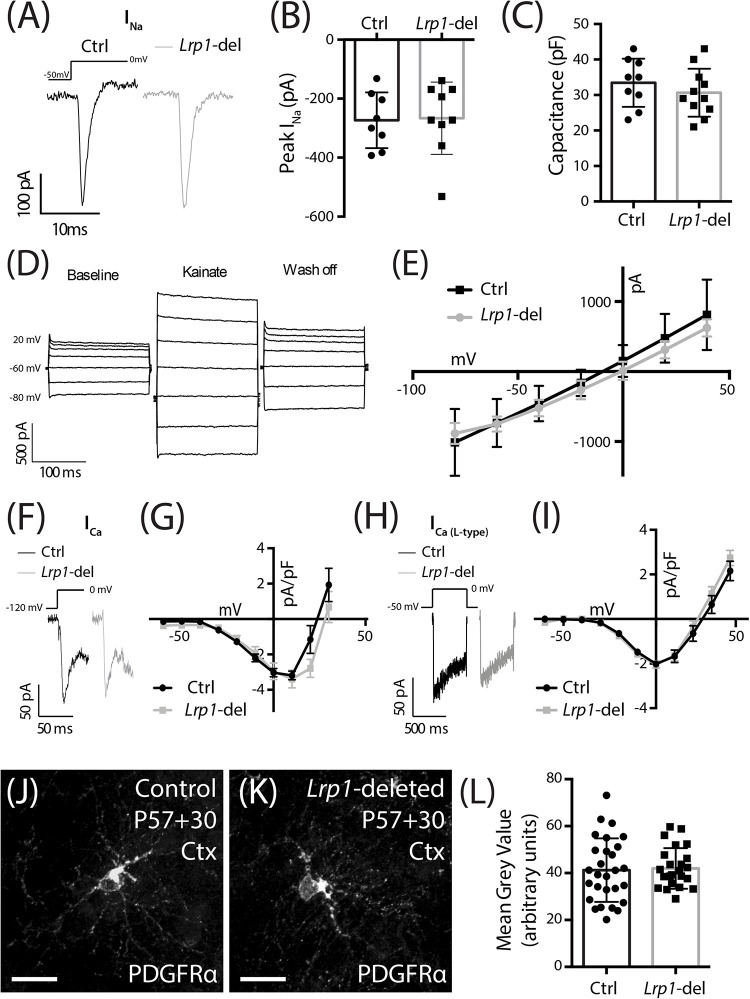
LRP1 does not alter functional NaV, VGCC, or AMPA/kainate receptor expression, or total PDGFRα expression in OPCs. **(A)** Representative traces of voltage-gated sodium channels currents (I_Na_) evoked in GFP^+^ OPCs in the motor cortex of P57+30 control (*Pdgfr*α*-histGFP :: Lrp1^*fl/fl*^*) and *Lrp1*-deleted (*Pdgfr*α*-CreER^*TM*^ :: Pdgfr*α*-histGFP :: Lrp1^*fl/fl*^)* mice. **(B)** Quantification of peak inward I_Na_ (*n* ≥ 8 GFP^+^ OPCs analyzed from *n* = 3 mice per genotype; unpaired *t*-test, *p* = 0.8). **(C)** Quantification of cell capacitance (*n* ≥ 9 GFP^+^ OPCs analyzed from *n* = 3 mice per genotype; unpaired *t*-test, *p* = 0.9). **(D)** Representative trace from a control GFP^+^ OPC responding to the bath application of 100 μM kainate. **(E)** The current density-voltage relationship of AMPA/kainate receptors in control (*n* = 3 GFP^+^ OPCs) and *Lrp1*-deleted (*n* = 3 GFP^+^ OPCs) cells [mean ± SEM; 2-way repeated measures ANOVA: *Genotype F* (1, 28) = 0.91, *p* = 0.3; *Voltage F* (6, 28) = 31.3, *p* < 0.0001; *Interaction F* (6, 28) = 0.25, *p* = 0.9]. **(F)** Representative traces show the fast inactivating leak subtracted I_Ca_ evoked in GFP^+^ OPCs in response to a depolarizing step. **(G)** The current density-voltage relationship for the leak subtracted I_Ca_ (peak amplitude) recorded from control cells (dark circles, *n* = 11 GFP^+^ OPCs across *n* = 3 mice) and *Lrp1*-deleted cells (gray squares, *n* = 10 GFP^+^ OPCs across *n* = 3 mice) [mean ± SEM; 2-way repeated measures ANOVA: *Genotype F* (1, 190) = 2.85, *p* = 0.09; *Voltage F* (9, 190) = 23.5, *p* < 0.0001; *Interaction F* (9, 190) = 1.14, *p* = 0.3]. **(H)** Representative traces show the leak subtracted I_Ca_ L-type evoked in GFP^+^ OPCs in response to a depolarizing step. **(I)** The current density-voltage relationship for leak subtracted I_Ca_ L-type (mean sustained current) recorded from control cells (dark circles, *n* = 7 GFP^+^ OPCs across *n* = 3 mice) and *Lrp1*-deleted cells (gray squares, *n* = 11 GFP^+^ OPCs across *n* = 3 mice) [mean ± SEM; 2-way repeated measures ANOVA: *Genotype F* (1, 176) = 1.03, *p* = 0.3; *Voltage F* (10, 176) = 66.8, *p* < 0.0001; *Interaction F* (10, 176) = 0.62, *p* = 0.8]. **(J,K)** Compressed *z*-stack confocal image of PDGFRα^+^ OPCs in the motor cortex (Ctx) of P57+30 control and *Lrp1-*deleted mice. **(L)** The mean gray value of PDGFRα staining for individual OPCs measured in the motor cortex of P57+30 control and *Lrp1-*deleted mice (mean ± SD; *n* ≥ 24 OPCs measured across *n* = 3 mice per genotype; unpaired *t*-test, *p* = 0.8). Scale bars represent 17 μm.

LRP1 has the potential to influence a number of signaling pathways that directly or indirectly regulate oligodendrogenesis. The conditional deletion of *Lrp1* from neurons *in vitro* and *in vivo* increases AMPA receptor turnover and reduces expression of the GluA1 subunit of the AMPA receptor (Gan et al., 2014). Adult OPCs express AMPA receptors (Gallo et al., 1996; Gudz, 2006; Zonouzi et al., 2011) that enhance the survival of premyelinating oligodendrocytes during development (Kougioumtzidou et al., 2017) and glutamatergic signaling regulates OPC proliferation, differentiation (Gallo et al., 1996; Fannon et al., 2015) and migration (Gudz, 2006). To determine whether LRP1 regulates AMPA receptor signaling in OPCs we obtained whole cell patch clamp recordings from GFP-labeled OPCs in the motor cortex of P57+30 control (*Lrp1^*fl/fl*^ :: Pdgfr*α*-histGFP*) and *Lrp1*-deleted (*Pdgfr*α*-CreER^TM^ :: Lrp1^*fl/fl*^ :: Pdgfr*α*-histGFP*) mice (Figure 6). OPCs elicit a large inward voltage-gated sodium current (I_Na_) in response to a series of voltage-steps (Figure 6A). We found that I_Na_ amplitude (Figure 6B) and OPC capacitance (approximation of cell size; Figure 6C) were not affected by LRP1 expression. AMPA receptors were subsequently activated by the bath application of 100 μm kainate, which evoked a large depolarizing current in control and *Lrp1*-deleted OPCs (Figures 6D,E). The amplitude of the evoked current was equivalent for control and *Lrp1*-deleted OPCs across all voltages examined (Figure 6E), suggesting that *Lrp1* deletion has no effect on the composition or cell-surface expression of AMPA/kainate receptors.

LRP1 regulates the cell surface expression and distribution of *N*-type voltage gated calcium channels (VGCC) by interacting with the α_2_δ subunit (Kadurin et al., 2017). In adult OPCs, the closely related *L*-type VGCCs reduce OPC proliferation in the motor cortex and corpus callosum (Pitman et al., 2019) and influence OL maturation *in vitro* (Cheli et al., 2015). The other major VGCCs expressed by OPCs are *T*-type VGCCs (Williamson et al., 1997; Fulton et al., 2010) which are activated at lower (hyperpolarized) voltages than *L*-type channels and inactivate quickly (transient). To determine whether the cell surface expression of VGCCs is altered following *Lrp1* deletion, we performed whole cell patch clamp electrophysiology and measured the current density (pA/pF) in OPCs from control and *Lrp1*-deleted mice (Figures 6F–I). We found that the VGCC current density was equivalent for OPCs in the motor cortex of control and *Lrp1*-deleted mice (Figures 6F,G). The current density was also equivalent between OPCs from control and *Lrp1-*deleted mice when measured currents were elicited selectively through *L*-type VGCCs (Figures 6H,I), indicating that LRP1 does not influence *L*- or *T*-type VGCC expression in adult OPCs.

Tissue plasminogen activator (tPA) is an LRP1 ligand (Bu et al., 1992; Orth et al., 1992) and its addition to astrocytic cultures increases PDGF-CC cleavage and activation (Su et al., 2008). While PDGF-CC is a ligand of PDGFRα, a key receptor regulating OPC proliferation, survival and migration (Noble et al., 1988; Richardson et al., 1988; Pringle et al., 1989), increased mitogenic stimulation would not account for LRP1 reducing adult OPC proliferation. In other cell types, LRP1 has instead been shown to influence the cell surface expression of PDGFRβ (Takayama et al., 2005; Muratoglu et al., 2010), a receptor that is closely related to PDGFRα. When performing immunohistochemistry using an antibody against the intracellular domain of PDGFRα, it is not possible to specifically quantify the cell surface expression of PDGFRα in OPCs with and without LRP1, however, we were able to quantify PDGFRα expression (mean gray value; Figures 6J–L), and determined that LRP1 did not influence total PDGFRα expression.

The low-density lipoprotein receptor related protein 2 (LRP2) is a large cell surface receptor that is closely related to LRP1, with a number of common ligands (Spuch et al., 2012). LRP2 can increase the proliferation of neural precursor cells in the subependymal zone (Gajera et al., 2010) and the proliferation and survival of skin cancer cells (Andersen et al., 2015), however, it is unclear whether cells of the OL lineage express LRP2 (Cahoy et al., 2008; Zhang et al., 2014; Hrvatin et al., 2018), or whether *Lrp1* deletion could alter LRP2 expression. We examined this possibility by performing immunohistochemistry on coronal brain cryosections from P57+30 control and *Lrp1*-deleted mice to detect LRP2 and PDGFRα or ASPA (Supplementary Figure 2). We determined that LRP2 is not expressed by OPCs or OLs in mice of either genotype, despite the robust expression of LRP2 by Iba1^+^ microglia (Supplementary Figure 2). These data indicate that compensation from LRP2 or a change in LRP2 expression by OPCs is not responsible for the elevated OPC proliferation and differentiation observed in *Lrp1*-deleted mice.

### *Lrp1* Deletion Does Not Alter EdU Incorporation by OPCs *in vitro*

**FIGURE 7:**
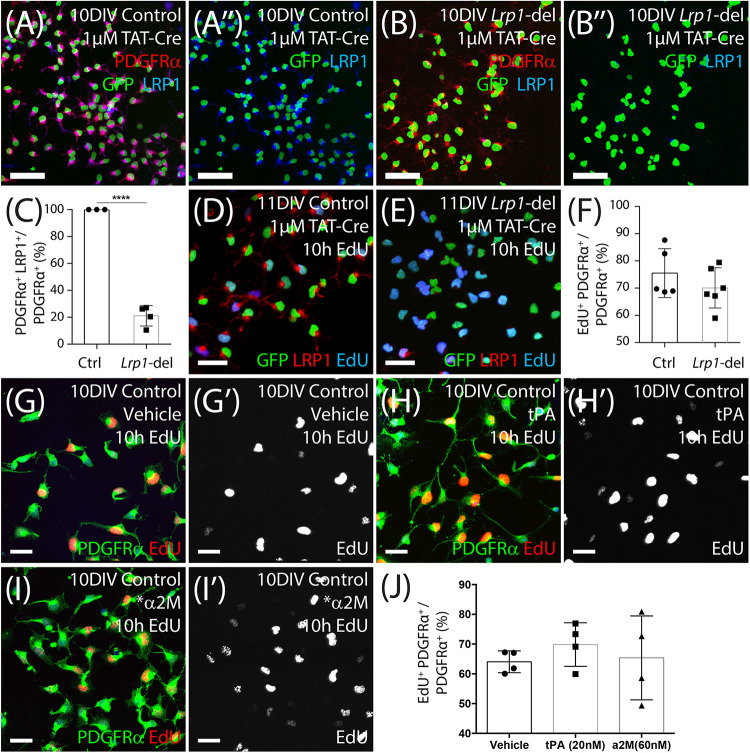
LRP1 does not affect OPC proliferation *in vitro*. **(A–B″)** Tat-Cre-treated OPCs cultured from the cortex of early postnatal control (*Pdgfr*α*-histGFP*) and *Lrp1*-deleted (*Pdgfr*α*-histGFP :: Lrp1^*fl/fl*^*) mice were immunolabeled to detect PDGFRα (red), LRP1 (blue), and GFP (green). **(C)** Quantification of the proportion (%) of control and *Lrp1*-deleted OPCs that express LRP1 48 h after TAT-Cre treatment (mean ± SEM, *n* ≥ 3 independent cultures per genotype; unpaired *t*-test, ****p < 0.0001). **(D,E)** Tat-Cre-treated OPCs from control and *Lrp1*-deleted mice exposed to EdU for 10 h and immunolabeled to detect GFP (green), LRP1 (red), and EdU (blue). **(F)** Quantification of the proportion (%) of control and *Lrp1*-deleted OPCs that become EdU-labeled over a 10-h period (mean ± SEM, *n* ≥ 5 independent cultures per genotype; unpaired *t*-test, *p* = 0.3). **(G–I′)** Compressed confocal *z*-stack showing OPCs cultured from control mice that were exposed to EdU and either vehicle (MilliQ water; **G,G′**), 20 nM tPa **(H,H′)** or 60 nM *α2M **(I,I′)** for 10 h, before being processed to detect EdU (red) and PDGFRα (green). **(J)** Quantification of the proportion (%) of control OPCs that incorporated EdU when treated with vehicle, tPa or *α2M for 10 h (mean ± SEM, *n* ≥ 4 independent cultures; 1-way ANOVA: *Treatment F* (2, 9) = 0.42, *p* = 0.66). Scale bars represent 17 μm. DIV = days *in vitro*; tPA = tissue plasminogen activator; *α2M = activated α-2 macroglobulin.

Our data suggest that in the healthy adult mice, *Lrp1* deletion either increases OPC proliferation which then results in an increased number of newborn OLs, or increases OPC differentiation which subsequently triggers a homeostatic increase in OPC proliferation to maintain the OPC population. Previous studies have shown that *Lrp1* deletion enhances the proliferation of retinal endothelial cells (Mao et al., 2016) and reduces the proliferation of cells within embryonic mouse neurospheres (Safina et al., 2016), while the activation of LRP1 by tPA enhances the proliferation of interstitial fibroblasts (Lin et al., 2010). To determine whether LRP1 directly suppresses OPC proliferation, we generated primary OPC cultures from the cortex of P0–P5 control (*Pdgfrα-hGFP)* or *Lrp1*-deleted (*Pdgfrα-hGFP: Lrp1^*fl/fl*^*) mice. To delete *Lrp1 in vitro*, we used a cell-permeable form of Cre-recombinase, TAT-Cre, which is a fusion peptide of Cre-recombinase and the HIV cell-penetrating TAT peptide. After 7 days *in vitro* (DIV), OPCs were incubated with 1 μM TAT-Cre for 90 min and LRP1 expression was determined at 9 DIV by performing immunocytochemistry to detect PDGFRα (red), GFP (green), and LRP1 (blue) (Figures 7A,B). Following Tat-Cre treatment all OPCs cultured from control mice expressed LRP1, however, only ∼21% of PDGFRα^+^ OPCs cultured from *Lrp1*-deleted mice retained LRP1 expression (Figure 7C). At the same time-point, additional control and *Lrp1*-deleted OPC cultures were exposed to EdU to label all cells that entered the S-phase of the cell cycle over a 10-h period. By performing immunocytochemistry to detect GFP (green), LRP1 (red) and EdU (Figures 7D,E), we found that LRP1 expression did not influence OPC proliferation *in vitro*, as the fraction of OPCs that were EdU^+^ was equivalent in control and *Lrp1-*deleted cultures (Figure 7F).

To determine whether LRP1 activation by ligands could directly influence OPC proliferation, we added vehicle (MilliQ water) or the LRP1 ligands tPA (20 nM) or activated α-2 macroglobulin (^∗^α2M; 60 mM) to OPC primary cultures for 10 h, along with EdU (Figures 7G–J). By performing immunocytochemistry to detect PDGFRα (green) and EdU (red), we determined that the proportion of OPCs that became EdU-labeled did not change with the addition of tPA or ^∗^α2M (Figure 7J), indicating that LRP1 activation by these ligands is unable to acutely modify OPC proliferation *in vitro*.

### *Lrp1* Deletion Increases the Proportion of OPCs That Differentiate *in vitro*

**FIGURE 8:**
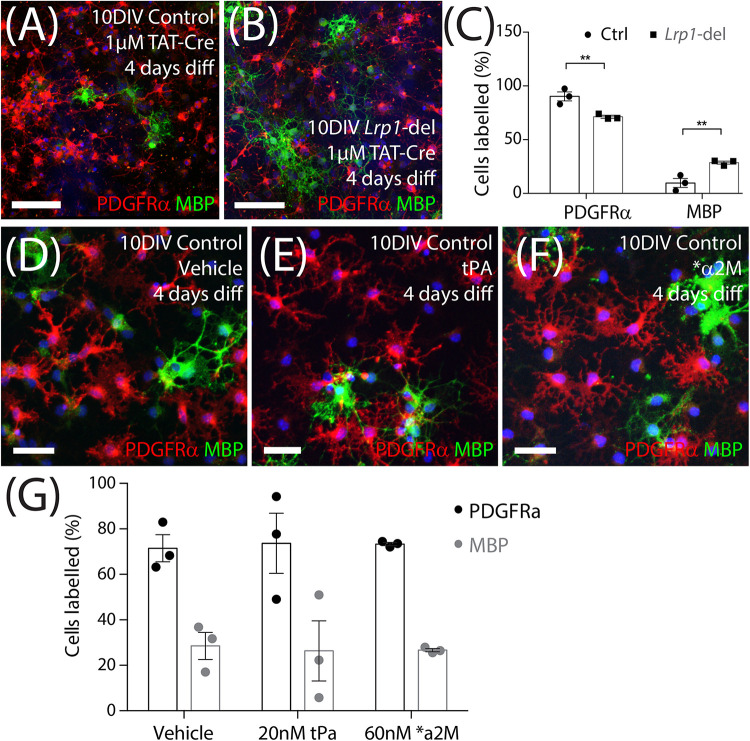
*Lrp1* deletion increases OPC differentiation *in vitro*. **(A,B)** Control and *Lrp1*-deleted OPCs were exposed to Tat-Cre, cultured for a further 48-h, and then transferred to differentiation medium for 4 days. Compressed confocal *z*-stack showing differentiated control and *Lrp1*-deleted (*Lrp1*^*fl/fl*^) OPC cultures immunolabeled to detect OPCs (PDGFRα, red), OLs (MBP, green), and all cell nuclei (Hoechst 33342, blue). **(C)** Quantification of the proportion (%) of cells that were PDGFRα^+^ OPCs or MBP^+^ OLs in control and *Lrp1-deleted* cultures [mean ± SEM for *n* = 3 independent cultures per genotype; 2-way ANOVA: *Cell type F* (1, 8) = 212, *p* < 0.0001; *Genotype F* (1, 8) = 0.001, *p* = 0.97; *Interaction F* (1, 8) = 19.9, *p* = 0.02]. Bonferroni multiple comparisons: **p = 0.005. **(D–F)** Compressed confocal *z*-stack shows OPCs from control mice that were transferred into differentiation medium and exposed to vehicle (MilliQ water; **D**), tPa **(E)**, or *α2M **(F)** for 4 days before being immunolabeled to detect OPCs (PDGFRα, red), OLs (MBP, green), and all cell nuclei (Hoechst 33342, blue). **(G)** Quantification of the proportion (%) of OPCs that became PDGFRα^+^ OPCs or MBP^+^ OLs after 4 days in differentiation medium with vehicle, tPa or *α2M [mean ± SEM, *n* = 3 independent cultures per treatment; 2-way ANOVA: *Cell type F* (1, 12) = 44.6, *p* < 0.0001; *Treatment F* (2, 10) = 0, *p* = 1; *Interaction F* (2, 12) = 0.03, *p* = 0.97]. tPa = tissue plasminogen activator; *α2M = activated α2 macroglobulin. Scale bars represent 34 μm **(A,B)** or 17 μm **(D–F)**.

*In vitro*, OPCs can be triggered to differentiate by withdrawing the mitogen PDGF-AA and providing triiodothyronine (T3) in the culture medium. To determine whether *Lrp1* deletion can enhance OPC differentiation, TAT-Cre-treated control and *Lrp1*-deleted OPCs were transferred into differentiation medium for 4 days before they were immuno-labeled to detect PDGFRα^+^ OPCs (red) and MBP^+^ OLs (green) (Figures 8A,B). We determined that the proportion of cells that were PDGFRα^+^ OPCs was reduced in the *Lrp1*-deleted cultures, while the proportion of cells that were MBP^+^ OLs was significantly increased compared with control cultures (Figure 8C).

To determine whether the ligand activation of LRP1 was sufficient to suppress OPC differentiation, OPC primary cultures were transferred into differentiation medium containing vehicle (MilliQ water), tPA (20 nM) or ^∗^α2M (60 nM) for 4 days. By performing immunocytochemistry to detect PDGFRα^+^ OPCs and MBP^+^ OLs (Figures 8D–F) we found that the activation of LRP1 by tPA or ^∗^α2M had no impact on the proportion of cells that differentiated over time (Figure 8G). As ApoE4 produces an LRP1-dependent increase in Erk and Akt phosphorylation to increase the number of O4^+^ OLs produced by differentiating embryonic mouse neurospheres (Safina et al., 2016), we also added vehicle (MilliQ water) or ApoE4 (203 nM) to a distinct set of proliferating and differentiating OPC cultures. We report that ApoE4 had no effect on the proportion (%) of OPCs that incorporated EdU (vehicle: 56.2 ± 11.5%; ApoE4: 57.2 ± 7.3%, mean ± SEM for *n* = 4 independent cultures; paired *t*-test *t* = 0.2, *p* = 0.8). Furthermore, the proportion (%) of cells that were MBP^+^ OLs (vehicle, 29.1 ± 4.3% and ApoE4, 35.9 ± 3.4%) after 4 days of differentiation, was equivalent in vehicle and ApoE4-treated cultures [mean ± SEM for *n* = 4 independent cultures; paired *t*-test *t* = 1.0, *p* = 0.4]. These data suggest that the LRP1 ligands tPA, α2M and ApoE4, do not regulate OPC differentiation *in vitro*.

### *Lrp1* Deletion Increases the Number of Newborn Mature OLs Added to the Corpus Callosum During Cuprizone Feeding

**FIGURE 9:**
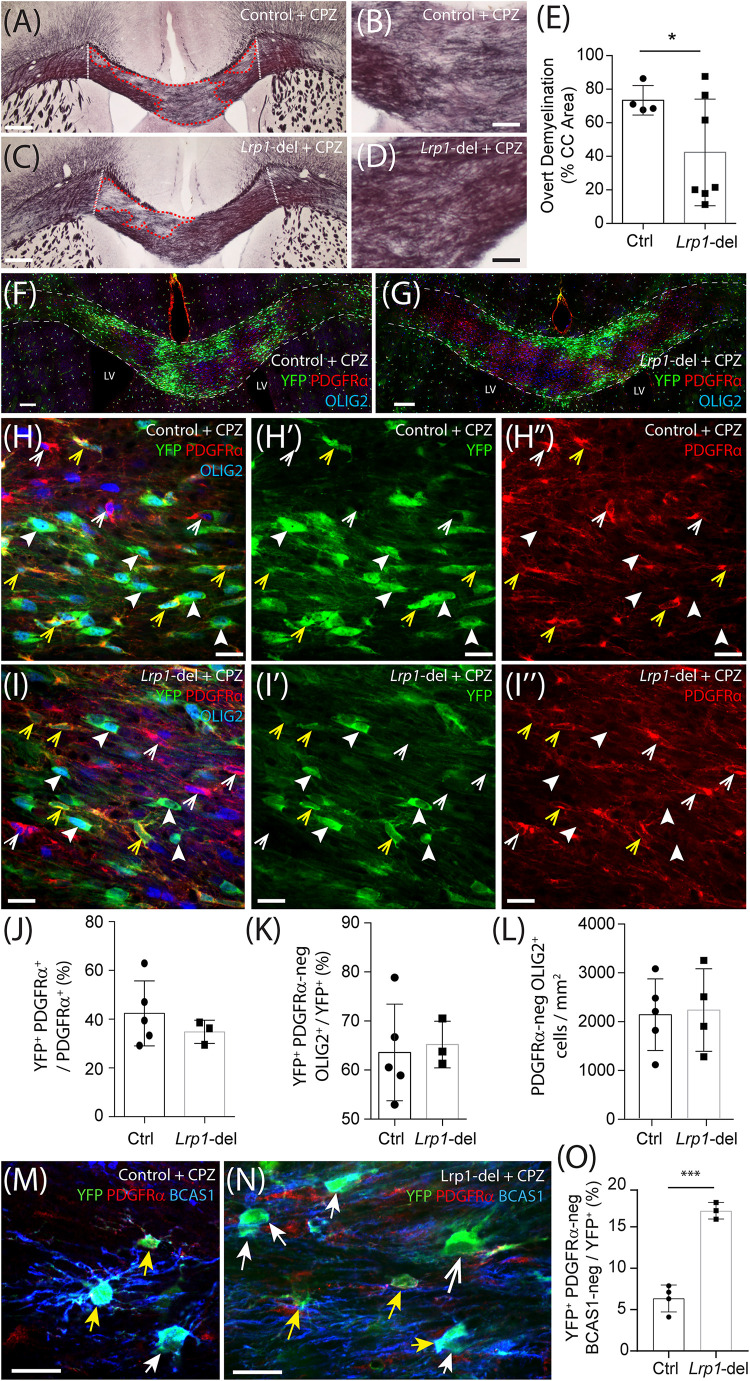
*Lrp1* deletion increases the number of mature OLs added to the mouse corpus callosum during cuprizone feeding. **(A–D)** Young adult control (*Pdgfr*α*-CreER^*TM*^ :: Rosa26-YFP*) and *Lrp1*-deleted (*Pdgfr*α*-CreER^*TM*^ :: Rosa26-YFP :: Lrp1^*fl/fl*^*) mice were fed a diet containing 0.2% cuprizone for 5 weeks. At the end of 5 weeks, coronal brain sections were collected and stained with black-gold to visualize myelin in the corpus callosum. Dotted white lines define the lateral limits of the region of the corpus callosum analyzed. Red dashed lines denote areas of overt demyelination. **(E)** The proportion (% area) of the corpus callosum with faint or absent black-gold staining (overt demyelination) in cuprizone-fed control and *Lrp1*-deleted mice (mean ± SD, *n* ≥ 4 mice per genotype; unpaired with Welch’s correction, *p = 0.04). **(F–I″)** Compressed confocal *z*-stack shows the corpus callosum in cuprizone-fed control and *Lrp1*-deleted mice, immunolabeled to detect YFP (green), PDGFRα (red), and OLIG2 (blue). White dashed lines indicate the dorsal and ventral boundaries of the corpus callosum; yellow arrowheads indicate YFP^+^ PDGFRα^+^ parenchymal OPCs; solid white arrowheads indicate YFP^+^ PDGFRα-negative newborn OLs; white arrowheads indicate YFP-negative PDGFRα^+^ stem cell-derived OPCs. **(J)** Quantification of the proportion (%) of OPCs in the corpus callosum of cuprizone-fed control and *Lrp1*-deleted mice that are YFP^+^, PDGFRα^+^ parenchymal OPCs (mean ± SD, *n* ≥ 3 mice per genotype; unpaired *t*-test, *p* = 0.4). **(K)** Quantification of the proportion of YFP^+^ cells in the corpus callosum of cuprizone-fed control and *Lrp1*-deleted mice that are PDGFRα-negative OLIG2^+^ newborn OLs (mean ± SD, *n* ≥ 3 mice per genotype; unpaired *t*-test, *p* = 0.9). **(L)** Quantification of the density of PDGFRα-negative OLIG2^+^ OLs in the corpus callosum of cuprizone-fed control and *Lrp1*-deleted mice (mean ± SD, *n* ≥ 4 mice per genotype; unpaired *t*-test, *p* = 0.9). **(M,N)** Single *z*-plane confocal images showing the corpus callosum of cuprizone-fed control and *Lrp1-*deleted mice stained to detect YFP (green), PDGFRα (red), and BCAS1 (blue). Solid yellow arrows indicate parenchymal OPCs (YFP^+^ PDGFRα^+^ ± BCAS1). Solid white arrows indicate newborn premyelinating OLs derived from parenchymal OPCs (YFP^+^ PDGFRα-neg BCAS1^+^). Large white arrow indicates newborn mature OL derived from parenchymal OPCs (YFP^+^ PDGFRα-neg BCAS1-neg). **(O)** Quantification of the proportion of YFP^+^ cells in the corpus callosum of cuprizone-fed control and *Lrp1-*deleted mice that are YFP^+^ PDGFRα-neg BCAS1-neg newborn mature OLs (mean ± SD for *n* ≥ 3 mice per genotype; unpaired *t*-test, *t* = 9.8, ***p = 0.0002). Scale bars represent 150 μm **(A,C)**, 30 μm **(B,D)**, 100 μm **(F,G)**, 17 μm **(H,I)**, or 20 μm **(M,N)**. LV, lateral ventricle.

As *Lrp1* deletion enhances OL addition to the healthy adult mouse brain, we next aimed to determine whether the deletion of *Lrp1* from adult OPCs could similarly increase OL generation in response to OL loss and demyelination. Control (*Pdgfr*α*-CreER^TM^ :: Rosa26-YFP)* and *Lrp1*-deleted (*Pdgfr*α-*CreER^TM^ :: Rosa26-YFP :: Lrp1^*fl/fl*^*) mice received tamoxifen by oral gavage at P57 and at P57+7 were transferred onto a diet containing 0.2% (w/w) cuprizone for 5 weeks to induce significant OL loss, demyelination of the corpus callosum and oligodendrogenesis. After 5 weeks of cuprizone feeding, control and *Lrp1*-deleted mice were perfusion fixed and coronal brain cryosections processed to detect myelin by black-gold staining (Figure 9). We identified and measured areas of overt demyelination in the corpus callosum of control and *Lrp1*-deleted mice (Figures 9A–D) and found that this represented a smaller proportion of the corpus callosum in *Lrp1*-deleted mice compared with controls (Figure 9E).

When EdU was delivered via the drinking water from week 2 to week 5 of cuprizone feeding, it was incorporated into the vast majority of OLIG2^+^ cells in the corpus callosum of control and *Lrp1*-deleted mice (Supplementary Figure 3). Despite being newborn cells, many of the PDGFRα^+^ OPCs (red) and PDGFRα-negative OLIG2^+^ OLs (blue) within the corpus callosum of control and *Lrp1*-deleted mice did not co-label with YFP (green) (Figures 9F,G), indicating that these cells were not derived from the YFP^+^ parenchymal OPC population. Following cuprizone-induced demyelination, both parenchymal OPCs (YFP-labeled) and neural stem cell-derived OPCs (YFP-negative) contribute to OL replacement and remyelination (Xing et al., 2014). After cuprizone feeding the proportion of OPCs that were YFP^+^ parenchymal OPCs was equivalent in the corpus callosum of control and *Lrp1*-deleted mice (Figure 9J). As total OPC density was also unaffected by genotype (838 ± 165 OPCs/mm^2^ in control and 740 ± 134 OPCs/mm^2^ in *Lrp1*-deleted corpus callosum; mean ± SD for *n* = 5 control and *n* = 3 *Lrp1-*deleted mice; unpaired *t*-test, *p* = 0.67), the expression of LRP1 by parenchymal OPCs does not appear to influence OPC production by neural stem cells.

Following demyelination, YFP^+^ parenchymal OPCs present in the corpus callosum of *Lrp1-*deleted mice lacked LRP1, however, the YFP-negative neural stem cell-derived OPCs had intact LRP1 expression (Supplementary Figure 3). Surprisingly, YFP^+^ parenchymal OPCs no longer generated more OLs in *Lrp1*-deleted mice compared to controls, as 60 ± 15% of YFP^+^ cells were PDGFRα-negative OLIG2^+^ newborn OLs in the corpus callosum of control mice and 65 ± 5% of YFP^+^ cells were PDGFRα-negative OLIG2^+^ newborn OLs in the corpus callosum of *Lrp1*-deleted mice (Figure 9K; mean ± SD for *n* = 5 control and *n* = 3 *Lrp1*-deleted mice). As total OL density was also equivalent in the corpus callosum of control and *Lrp1*-deleted mice (Figure 9L), a change in oligodendrogenesis did not appear to account for *Lrp1*-deleted mice having less overt demyelination. However, by performing immunohistochemistry to detect YFP, the OPC marker PDGFRα, and Breast Carcinoma Amplified Sequence 1 (BCAS1), a protein expressed by some OPCs and all pre-myelinating OLs (Fard et al., 2017; Ferreira et al., 2020), we found that the fraction of YFP^+^ cells that were mature OLs (YFP^+^ PDGFRα-neg BCAS1-neg) was increased in the corpus callosum of *Lrp1*-deleted mice compared to controls (Figures 9M–O). These data suggest that LRP1 expression by adult OPCs impairs newborn OL maturation in the cuprizone-injured CNS.

## Discussion

Within the OL lineage, LRP1 is highly expressed by OPCs and is rapidly down-regulated upon differentiation (Cahoy et al., 2008; Zhang et al., 2014; Auderset et al., 2016a; Fernandez-Castaneda et al., 2019), suggesting that LRP1 regulates the function or behavior of the progenitor cells. As LRP1 can signal in a number of different ways (Lillis et al., 2008; Auderset et al., 2016b; Bres and Faissner, 2019) and has been shown to influence cellular behaviors relevant to OPCs, such as proliferation, differentiation (Boucher et al., 2007; Mao et al., 2016; Safina et al., 2016) and migration (Mantuano et al., 2010, 2015; Barcelona et al., 2013; Ferrer et al., 2016; Sayre and Kokovay, 2019), we took a conditional gene deletion approach to determine whether *Lrp1* influenced the behavior of adult mouse OPCs. We report that *Lrp1*-deletion produces a delayed increase in adult OPC proliferation and increases the number of OPCs that differentiate into mature, myelinating OLs in the motor cortex and corpus callosum. *Lrp1* deletion does not, however, alter the number or length of internodes produced by the newborn, myelinating OLs. Furthermore, during cuprizone-induced demyelination, *Lrp1* deletion does not influence the total number of newborn OLs added to the corpus callosum, but a larger proportion of the newborn cells are mature OLs.

### Does LRP1 Have a Different Effect on Developmental and Adult OPCs in the Healthy CNS?

New OLs are added to the adult mouse CNS throughout life (Dimou et al., 2008; Rivers et al., 2008; Kang et al., 2010; Zhu et al., 2011), and when we followed the fate of adult OPCs after *Lrp1* deletion, we observed a significant increase in the number of new OLs added to the corpus callosum and motor cortex at P57+30 and P57+45 (Figure 3). In the healthy adult mouse brain, there is a significant population of pre-myelinating OLs (Xiao et al., 2016; Fard et al., 2017) that are constantly turned over, as ∼78% of newly generated pre-myelinating OLs survive for less than 2 days (Hughes et al., 2018). However, *Lrp1* deletion did not simply increase the pool of premyelinating OLs, as deleting *Lrp1* from adult OPCs ultimately increased the number of newborn mature ASPA^+^ OLs added to the brain (Figure 4). Furthermore, by using LE-*Pdgfrα-CreER^*T*2^ :: Tau-mGFP* transgenic mice to visualize the full morphology of the newly generated OLs, we were able to confirm that *Lrp1* deletion effectively increased the number of newborn OLs that had myelinating morphology (Figure 5). The gross myelination profile of individual myelinating OLs appeared unaffected by LRP1 expression, as OLs in the cortex of *Lrp1*-deleted mice supported the same number of internodes of an equivalent length as those elaborated by newborn myelinating OLs in the cortex of control mice (Figure 5).

These data suggest that LRP1 expression by adult OPCs has the capacity to influence the number of OPCs that differentiate and survive as newborn, myelinating OLs in the healthy mouse brain, without significantly impacting their gross myelination profile. This was unexpected, as it suggests that LRP1 plays a different role in adult OPCs compared with developmental OPCs. In the developing mouse optic nerve, deleting *Lrp1* from cells of the OL lineage (*Olig2-Cre :: Lrp1^*fl/fl*^*) reduces the number of OLs produced and results in hypomyelination by P21 (Lin et al., 2017). As LRP1 is expressed by radial glia and neural stem cells (Hennen et al., 2013; Safina et al., 2016; Auderset et al., 2016a), and the *Olig2* gene promoter can be active in some neural stem cells and transiently active in some astrocytes (Marshall et al., 2005; Cai et al., 2007), it is possible that the *Olig2*-Cre transgenic mouse results in the unintended deletion of *Lrp1* from some neural stem cells. This would be expected to reduce OL generation, as cultured embryonic NSPCs lacking *Lrp1* produce fewer cells of the OL lineage (Hennen et al., 2013; Safina et al., 2016). However, a series of *in vitro* experiments suggest that the constitutive loss of *Lrp1* from OPCs in development, results in longer-lasting detrimental changes within the lineage. After 2 days of differentiation, primary brain OPCs derived from early postnatal *Olig1-Cre* and *Olig1-Cre :: Lrp1^*fl/fl*^* mice, have an equivalent level of *Myrf*, *Mbp*, and *CNPase* mRNA expression (Fernandez-Castaneda et al., 2019), Similarly, after 3 days in differentiation medium, OPCs from control and conditionally-deleted (*Olig2-Cre :: Lrp1^*fl/fl*^*) mice contain an equivalent number of NG2^+^ OPCs or CNPase^+^ OLs (Lin et al., 2017). By 5 days of differentiation, however, cultures from the conditionally-deleted mice contain significantly fewer PLP^+^ or MBP^+^ OLs and these cells had impaired myelin biogenesis (Lin et al., 2017). Sterol-regulatory element binding protein-2 expression was elevated and peroxisomal biogenesis factor-2 expression reduced in cultures generated from *Olig2-Cre :: Lrp*1^*fl*/*fl*^ mice, but the combined delivery of cholesterol and pioglitazone rescued this phenotype (Lin et al., 2017). Therefore, it is likely that LRP1 plays an important role in cholesterol homeostasis and peroxisome biogenesis in developmental OPCs (Lin et al., 2017). As *Lrp1* deletion did not disrupt the gross myelinating morphology of newborn OLs added to the adult mouse CNS, this function of LRP1 does not appear to be retained by adult OPCs. However, this could only be truly ruled out by performing an ultrastructural analysis of the newly elaborated myelin.

### Why Does *Lrp1* Deletion Have a Delayed Effect on OPC Proliferation in the Healthy Adult Mouse CNS?

At any one time, the majority of OPCs in the healthy adult mouse CNS are in the G_0_ phase of the cell cycle (Psachoulia et al., 2009). In young adulthood, all OPCs in the corpus callosum re-enter the cell cycle and divide at least once in a 10-day period, but a similar level of turnover takes ∼38 days for OPCs in the cortex (Young et al., 2013). In this study, we found that *Lrp1-*deletion increased the rate at which OPCs re-entered the cell cycle and became EdU-labeled, but the onset of this phenotype was not coincident with the loss of LRP1 (Figures 1, 2). More specifically, when EdU labeling commenced at P57+7, the proportion of OPCs that became EdU labeled over time was the same for control and *Lrp1*-deleted mice (Figure 2). However, when EdU delivery was delayed and instead commenced at P57+28, the proportion of OPCs that incorporated EdU was elevated in the corpus callosum or motor cortex of *Lrp1*-deleted mice compared with controls. It is feasible that LRP1 directly suppresses adult OPC proliferation. LRP1 is known to modulate the proliferation of other cell types (Boucher et al., 2007; Basford et al., 2009; Mao et al., 2016; Safina et al., 2016; Yang et al., 2018; Zucker et al., 2019), suppressing the hypoxia-induced proliferation of mouse and human retinal endothelial cells by regulating the activity of poly (ADP-ribose) polymerase-1 (PARP-1; Mao et al., 2016), and suppressing the proliferation of cultured mouse vascular smooth muscle cells by reducing PDGFRβ activity (Boucher et al., 2003; Basford et al., 2009). However, the inability of *Lrp1* deletion to acutely influence OPC proliferation *in vivo* or directly influence OPC proliferation *in vitro* (Figure 7; Lin et al., 2017), may suggest that LRP1 affects OPC proliferation indirectly.

**FIGURE 10:**
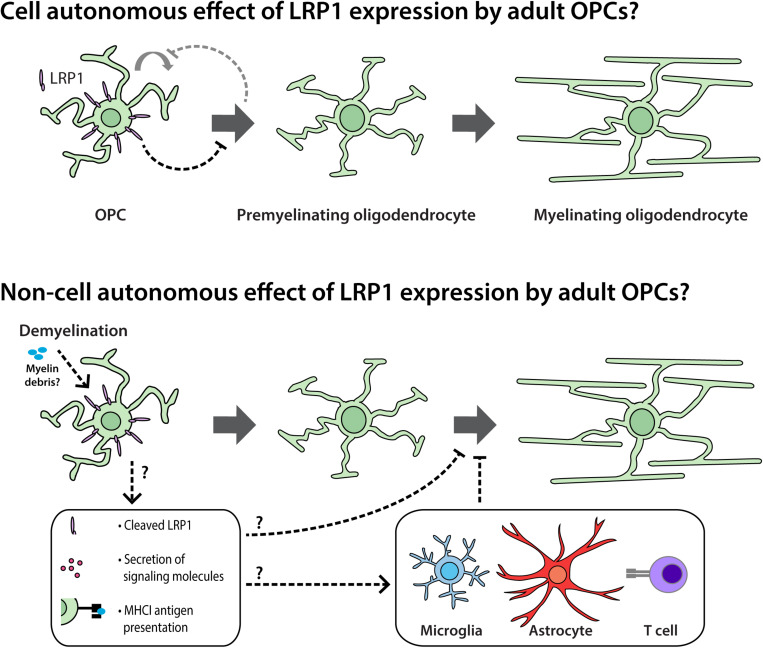
LRP1 signaling in adult OPCs may have cell autonomous and non-cell autonomous effects on myelination and remyelination. Cell autonomous: *in vivo*, LRP1 signaling by adult mouse OPCs reduces OPC proliferation and the number of newborn OLs added to the brain. As LRP1 suppresses OPC differentiation *in vitro*, we propose that LRP1 can directly suppress OPC differentiation. This cell autonomous effect would also be expected to reduce OPC proliferation, as these processes are closely and homeostatically linked *in vivo*. Non-cell autonomous: during cuprizone-induced demyelination, LRP1 expression by OPCs hinders OL maturation. This may be the result of a cell autonomous function of LRP1 in adult OPCs that has lasting effects during differentiation, but may also be the result of a less direct signaling mechanism. For example, the release of soluble LRP1 from the surface of OPCs; the LRP1-directed secretion of proteins from OPCs; or an LRP1-directed increase in OPC-mediated antigen presentation could influence the maturation of pre-myelinating OLs or the behavior of other cell types such as microglia, astrocytes or lymphocytes.

OPC proliferation is intimately linked to OPC differentiation *in vivo* (Hughes et al., 2013). As the number of new OLs detected in the adult mouse brain increases following the conditional deletion of *Lrp1* (Figures 3–5), it is possible that *Lrp1*-deletion directly enhances OPC differentiation and, in doing so, produces a homeostatic increase in OPC proliferation (see Figure 10). Further supporting this idea, the deletion of *Lrp1* from OPCs *in vitro*, directly increased the number of MBP^+^ OLs generated (Figure 8).

### How Does LRP1 Expression by Adult OPCs Affect OL Production in Response to an Injury?

As *Lrp1* deletion increased the number of new OLs detected in the healthy adult mouse brain, we predicted that *Lrp1* deletion would also enhance the number of new OLs produced in response to OL loss. However, OPCs can behave differently in the healthy versus injured CNS. It was previously reported that the delivery of tamoxifen to adult *CAG-CreER*^*TM*^ *:: Lrp*1^*fl*/*fl*^ mice, which reduced LRP1 expression by ∼50%, reduced the remyelination of a lysolecithin-induced callosal lesion (Hennen et al., 2013). This could have been the result of LRP1 loss from OPCs, microglia, astrocytes or neurons (May et al., 2004; Casse et al., 2012; Chuang et al., 2016; Yang et al., 2016; Liu et al., 2017; He et al., 2020), however, focal lesion repair was also impaired when *Lrp1* was conditionally deleted from adult OPCs (*Pdgfr*α*-CreER^TM^ :: Lrp1^*fl/fl*^* mice) (Hennen et al., 2013). While the level and specificity of *Lrp1* deletion was not reported in this study, these data suggest that LRP1 signaling in adult OPCs could improve myelin repair (Hennen et al., 2013).

We report that LRP1 experts an altogether different effect on adult OPCs following cuprizone-mediated OL loss and demyelination. By performing cre-lox lineage tracing to follow the fate of adult OPCs, we were able to quantify the number of new OLs added to the corpus callosum of control and *Lrp1*-deleted mice during cuprizone feeding. While the number of OPC-derived OLs was equivalent in control and *Lrp1*-deleted mice, a larger proportion of the new OLs had matured in the corpus callosum of *Lrp1*-deleted mice. Furthermore, the area of the corpus callosum showing overt demyelination was reduced in *Lrp1*-deleted mice compared with controls. Consistent with our findings, adult *Olig1-Cre* and *Olig1-Cre: Lrp1^*fl/fl*^* mice, subjected to 5 weeks of cuprizone feeding also had an equivalent number of OLs in the corpus callosum, however, within 3.5 days of cuprizone withdrawal, the corpus callosum of *Olig1-Cre: Lrp1^*fl/fl*^* mice contained more OLs than controls and had greater MBP coverage (Fernandez-Castaneda et al., 2019). Cuprizone-induced demyelination is a robust stimulus for OPC differentiation (Xing et al., 2014; Baxi et al., 2017) and could readily mask the lesser effect of *Lrp1* deletion on new OL addition. These data also suggest that LRP1 expression by OPCs impair newborn OL maturation, which could reflect a cell intrinsic function of LRP1 within the OL lineage or could reflect an alternative role for LRP1 allowing OPCs to regulate the maturation of adjacent maturing OLs by a less direct, perhaps inflammatory mechanism (see Figure 10).

Neuroinflammation impairs OL generation (Miron et al., 2011) and OPCs can modulate neuroinflammation, releasing cytokines in response to interleukin 17 receptor signaling (Wang et al., 2017). OPCs also express genes associated with antigen processing and presentation (Falcao et al., 2018; Kirby et al., 2019). While this was not investigated here, previous research has shown that LRP1 can bind and phagocytose myelin debris (Gaultier et al., 2009; Fernandez-Castaneda et al., 2013; Stiles et al., 2013) and that LRP1 expression by OPCs can influence the inflammatory nature of the remyelinating environment (Fernandez-Castaneda et al., 2019). RNA profiling of the remyelinating corpus callosum of *Olig1-Cre* and *Olig1-Cre: Lrp1^*fl/fl*^* mice, 3.5 days after cuprizone withdrawal, revealed that inflammatory gene expression was lower in *Olig1-Cre :: Lrp1^*fl/fl*^* mice (Fernandez-Castaneda et al., 2019). As LRP1 can be cleaved, it is possible that OPCs release soluble, cleaved LRP1 to enhance the inflammatory response of nearby microglia (Brifault et al., 2017, 2019). However, LRP1 may also facilitate antigen presentation by OPCs, as the deletion of *Lrp1* from OPCs reduces their expression of MHC class I antigen presenting genes in the corpus callosum of cuprizone-demyelinated mice and reduces the ability of OPCs to cross-present antigens to lymphocytes *in vitro* (Fernandez-Castaneda et al., 2019). Therefore, further research is required to fully elucidate the cell autonomous effect of LRP1 expression in OPCs and the capacity for LRP1 signaling on OPCs to influence the behavior of other nearby CNS cell types.

## Data Availability Statement

All datasets presented in this study are included in the article/Supplementary Material.

## Ethics Statement

The animal study was reviewed and approved by The University of Tasmania Animal Ethics Committee.

## Author Contributions

LA, KY, LF, and BT developed the project and wrote the manuscript. LA, CC, RP, and KP carried out the experiments. KY and LF obtained the funding. LA, CC, KP, and KY performed the statistical analyses and generated the figures. KY, LF, and BT provided supervision. All authors contributed to the article and approved the submitted version.

## Conflict of Interest

The authors declare that the research was conducted in the absence of any commercial or financial relationships that could be construed as a potential conflict of interest.
