# Supplementary material for: Low-Density Lipoprotein Receptor-Related Protein 1 (LRP1) Is a Negative Regulator of Oligodendrocyte Progenitor Cell Differentiation in the Adult Mouse Brain
Source: Front Cell Dev Biol. 2020 Nov 13;8:564351. doi: 10.3389/fcell.2020.564351 (PMC7691426; doi:10.3389/fcell.2020.564351)
Supplement: Supplementary file 1 [file Data_Sheet_1.docx]

Supplementary Material – Auderset et al.


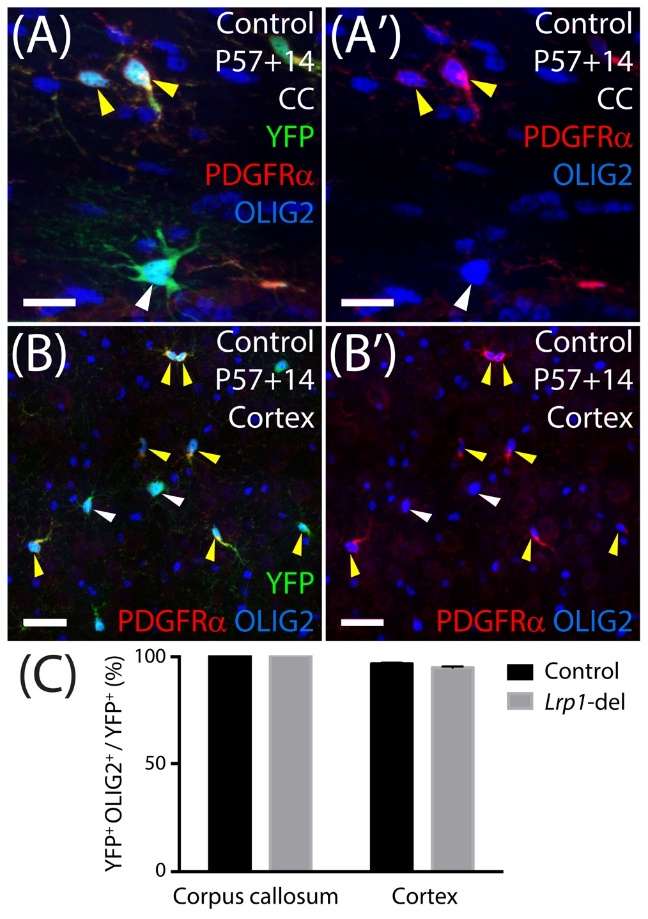


**Supplementary Figure 1: Essentially all YFP-labelled cells belong to the OL lineage**

(**A-B**) Confocal images from the corpus callosum (CC) and motor cortex (Cortex) of P57+14 control (*Pdgfrα-CreER^TM^ :: Rosa26YFP*) mice immunolabelled to detect OPCs (PDGFRα, red), YFP (green) and the transcription factor OLIG2 (blue). Solid yellow arrowheads indicate YFP^+^ OLIG2^+^ PDGFRα^+^ OPCs. Solid white arrowheads indicate YFP^+^ OLIG2^+^ PDGFRα-neg newborn OLs. (**C**) Quantification of the proportion (%) of YFP^+^ cells that express OLIG2 in the corpus callosum (100% ± 0% for control and 100% ± 0% for *Lrp1*-deleted) and motor cortex (96.1% ± 0.9% from control and 94.3% ± 1% for *Lrp1*-deleted mice) of P57+14 mice (mean ± SD, n= 3 mice per group). Scale bars represent: 34µm (A) or 17µm (B).

***
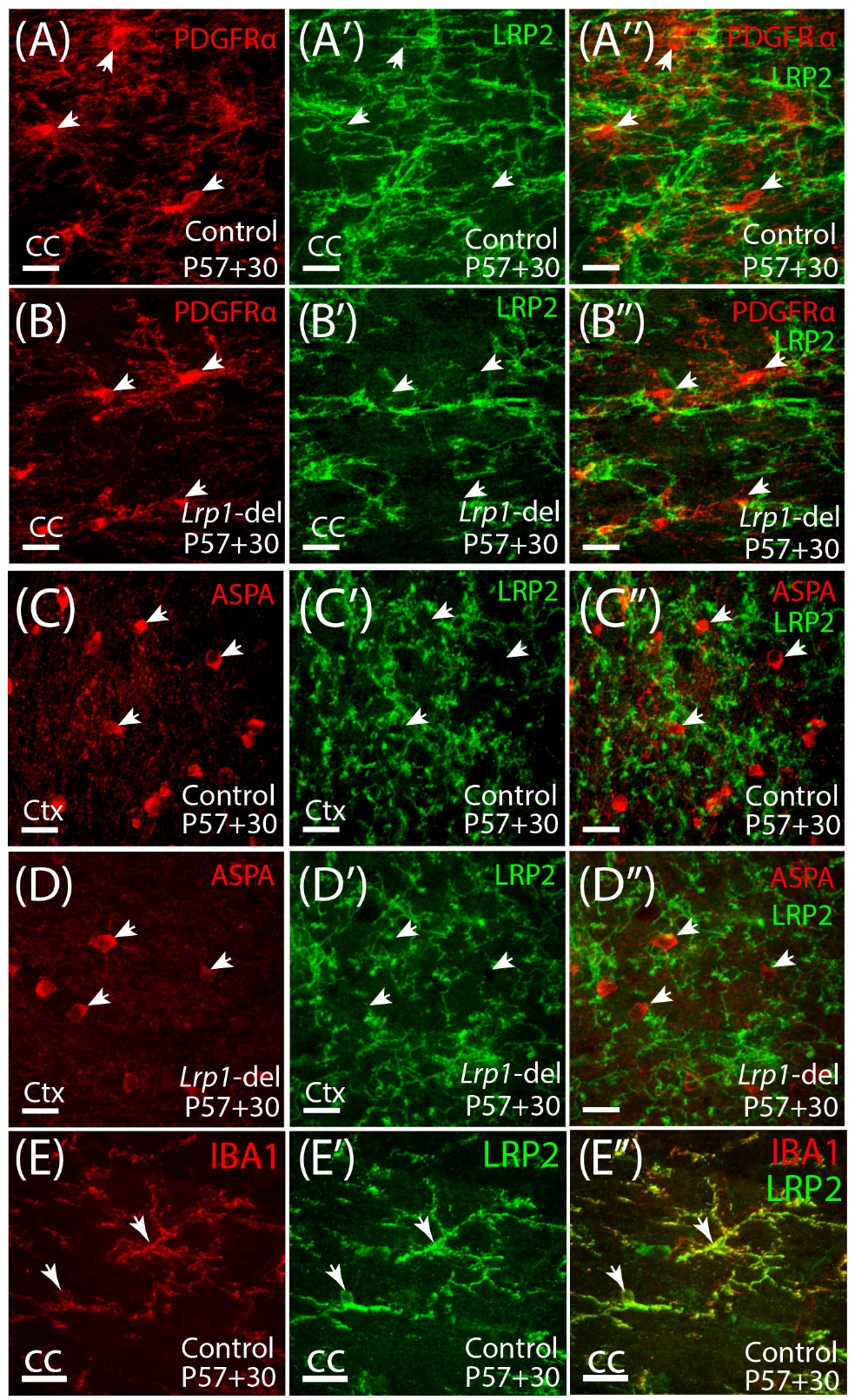
***

**Supplementary Figure 2: LRP2 is not expressed by OPCs or OLs, but is expressed by microglia**

(**A-B**) Compressed z-stack confocal images of the corpus callosum (CC) in a P57+30 control (*Pdgfrα-CreER^TM^)* and *Lrp1*-deleted (*Pdgfrα-CreER^TM^ :: Lrp1^fl/fl^*) mouse, immunolabelled to detect the OPC marker PDGFRα (red) and LRP2 (green). (**C-D**) Compressed z-stack confocal images of the motor cortex (Ctx) in a P57+30 control and *Lrp1*-deleted mouse, immunolabelled to detect the OL marker ASPA (red) and LRP2 (green). (**E**) Compressed z-stack confocal image of the CC in a P57+30 control mouse immunolabelled to detect the microglial marker IBA1 (green) and LRP2 (red). White arrows denote the location of the OPCs (A, B), OLs (C, D) or microglia (E). Scale bars represent 17µm.

**
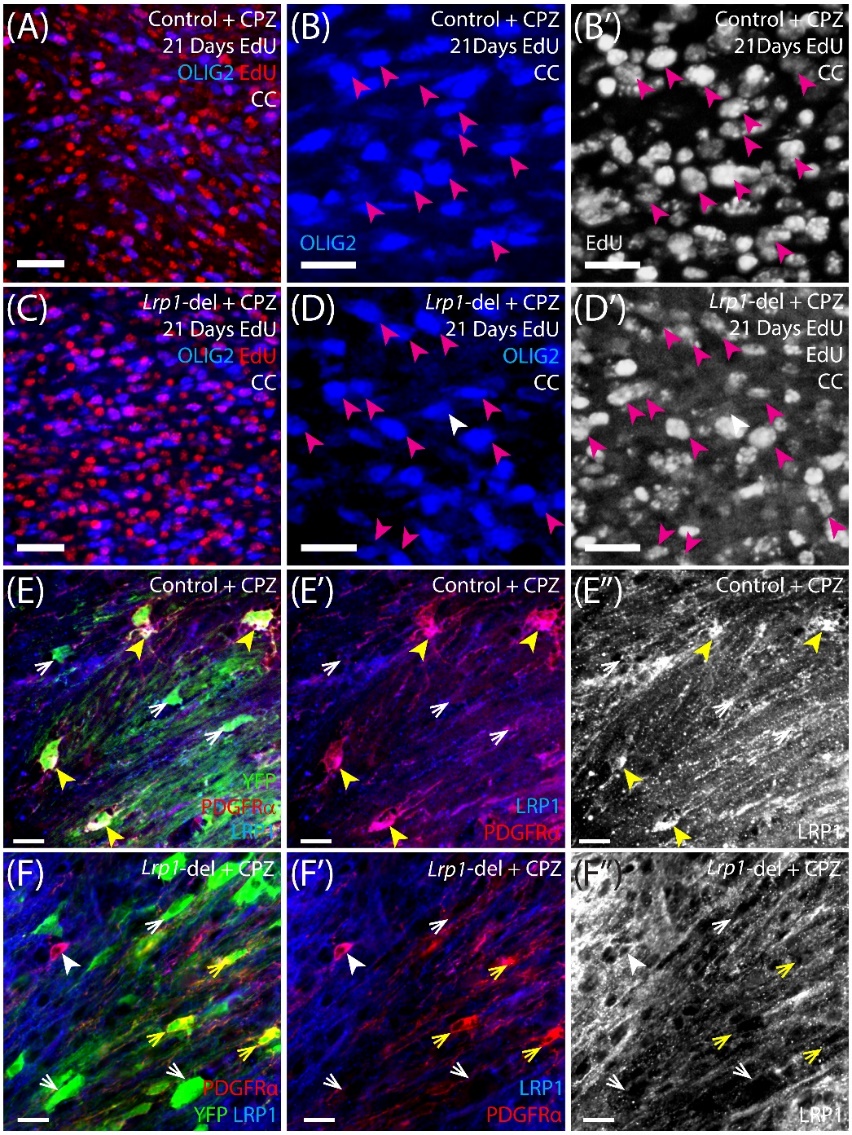
**

**Supplementary Figure 3: The vast majority of OLIG2^+^ cells present in the corpus callosum of cuprizone-fed control and *Lrp1-*deleted mice are newborn cells**

(**A-D)** Control (*Pdgfrα-CreER^TM^*) and *Lrp1*-deleted (*Pdgfrα-CreER^TM^ :: Lrp1^fl/fl^*) mice received cuprizone for 5 weeks, and also received EdU for the 3 finals weeks. Compressed z-stack confocal images show the corpus callosum of control (a, low magnification; b, high magnification) and *Lrp1*-deleted (c, low magnification; d, high magnification) mice labelled to detect the transcription factor OLIG2 (blue) and EdU (red). The vast majority of OLIG2^+^ cells in the corpus callosum of control (146 of 154 cells counted) and *Lrp1*-deleted mice (97 of 106 cells counted) were EdU^+^. Solid magenta arrowheads indicate example OLIG2^+^ EdU^+^ newborn cells. Solid white arrowheads indicate OLIG2^+^ EdU-neg cells. (**E-F**) Compressed z-stack confocal images of the corpus callosum of cuprizone-fed control (*Pdgfrα-CreER^TM^ :: Rosa26-YFP*) and *Lrp1*-deleted (*Pdgfrα-CreER^TM^ :: Rosa26-YFP :: Lrp1^fl/fl^*) mice immunolabelled to detect PDGFRα (red), YFP (green) and LRP1 (blue). YFP^+^ PDGFRα^+^ parenchymal OPCs in *Lrp1-*deleted mice lacked LRP1 (124 of 124 cells counted), however, the YFP-negative PDGFRα^+^ neural stem cell-derived OPCs had intact LRP1 expression (42 of 42 cells counted). Solid yellow arrowheads indicate YFP^+^ LRP1^+^ PDGFRα^+^ parenchymal OPCs in control tissue. Yellow arrows indicate YFP^+^ LRP1-negative PDGFRα^+^ parenchymal OPCs in *Lrp1-*deleted tissue. Solid white arrowheads indicate YFP-neg LRP1^+^ PDGFRα^+^ neural stem cell-derived OPCs. White arrows indicate YFP^+^ LRP1-neg PDGFRα-neg newborn OLs in control and *Lrp1*-deleted tissue. Scale bars represent 34µm (A, C and E-H) or 20µm (B, D). CC = corpus callosum.
